# A large dataset of annotated incident reports on medication errors

**DOI:** 10.1038/s41597-024-03036-2

**Published:** 2024-02-29

**Authors:** Zoie S. Y. Wong, Neil Waters, Jiaxing Liu, Shin Ushiro

**Affiliations:** 1 Graduate School of Public Health, St. Luke’s International University, 3-6-2 Tsukiji, Chuo-ku, Tokyo, 104-0045 Japan; 2 School of Medical Sciences, The University of Sydney, Camperdown, NSW 2006 Australia; 3 School of Statistics and Mathematics, Zhongnan University of Economics and Law, Nanhu Blvd, Wuhan, Hubei 430073 China; 4 Division of Patient Safety, Kyushu University Hospital, 3-1-1 Maidashi, Higashi-ku, Fukuoka, 812-8582 Japan; 5 Japan Council for Quality Health Care (JQ), 1-4-17, Toyo Bldg., Kandamisaki-cho, Chiyoda-ku, Tokyo, 101-0061 Japan

**Keywords:** Health services, Public health

## Abstract

Incident reports of medication errors are valuable learning resources for improving patient safety. However, pertinent information is often contained within unstructured free text, which prevents automated analysis and limits the usefulness of these data. Natural language processing can structure this free text automatically and retrieve relevant past incidents and learning materials, but to be able to do so requires a large, fully annotated and validated corpus of incident reports. We present a corpus of 58,658 machine-annotated incident reports of medication errors that can be used to advance the development of information extraction models and subsequent incident learning. We report the best F1-scores for the annotated dataset: 0.97 and 0.76 for named entity recognition and intention/factuality analysis, respectively, for the cross-validation exercise. Our dataset contains 478,175 named entities and differentiates between incident types by recognising discrepancies between what was intended and what actually occurred. We explain our annotation workflow and technical validation and provide access to the validation datasets and machine annotator for labelling future incident reports of medication errors.

## Background & Summary

Natural Language Processing is a promising technique for the extraction of information from medication related records to enhance medication safety^1^. However, only very few e-health systems can detect and learn from medical incidents. This poses a challenge to the ‘Patient Safety Incident Reporting and Learning Systems’ goal set by the WHO Global Patient Safety Action Plan 2021–2030^2,3^. Medication errors are a common and potentially avoidable cause of patient harm^4–6^ that, as stated in the third WHO Global Challenge on Patient Safety^7,8^ and the 2022 World Patient Safety Day, must be significantly reduced globally.

To capture clinical near misses and incidents, reporting systems are recommended by the Institute of Medicine and WHO. Millions of reports have been collected by national and institutional reporting systems across the world^9–12^. WHO has published the ‘Minimal Information Model for Patient Safety Incident Reporting and Learning Systems’^13^, a set of guidelines for standardised incident reporting. However, key information is often embedded within unstructured narrative accounts of the incident, e.g., the ‘free text’ section of reports. The number of narrative incident reports is beyond what can feasibly be reviewed and synthesised by humans, necessitating analysis by computers. Synthesising this plethora of varied free text into meaningful, standardised data that can guide learning and reduce medication errors remains challenging^2^. To guide the development of models capable of structuring this valuable free-text data into a structured format, a large, fully annotated, and validated corpus of incident reports of medication errors would be valuable.

To our knowledge, no such corpus is yet available, perhaps because the data within incident reports are usually proprietary to certain research groups/institutions and not openly accessible. The Japan Council for Quality Health Care (JQ) has collected more than 100,000 reports of adverse events and near misses, as part of their ‘Project to Collect Medical Near-Miss/Adverse Event Information’ (https://www.med-safe.jp/mpsearch/SearchReport.action). These free-text reports, published in the Japanese language and collected on a national scale since 2010, are openly available and could thus be used to create an annotated corpus of incident reports of medication errors.

Natural language processing and artificial intelligence (AI) are used to autonomously retrieve or extract information for the purpose of learning from incident reports^12,14–18^. Our earlier studies^14,19^ provided systematic methodologies for annotating and classifying incident reports of medication errors in a structured manner; the resulting high quality gold-standard data has been published^20^. In this study, we devised a data creation workflow and developed a machine annotator to create a large corpus of machine-annotated incident reports of medication errors, possessing useful drug-related concept and attribute extraction.

In total, we captured 478,175 medication error-related named entities from 58,568 incident reports of medication errors. For each incident report in the corpus, the relevant named entities have been identified, the discrepancy between what was intended and what actually occurred has been determined and what type of incident the report describes has been recognised. This corpus is the world’s largest publicly available body of annotated incident reports covering concepts and attributes (assertions) related to drug errors. Currently, the machine annotator was trained to detect medication error-related named entities within incident reports and was evaluated using incident reports. However, the machine annotator could be rescaled and applied to other document types, e.g., the free text found in electronic health records. This could be done via additional pretraining and finetuning processes and technical validation.

Furthermore, future studies may be conducted to examine how the human factors that contribute to error (e.g., distractions or workload) can be annotated systematically and added to this kind of dataset. Such knowledge would further enhance our potential to learn from incident reports. We envision the structuring of incident reports to ultimately transform incident learning in healthcare and translate recent advances in AI to improved patient safety. The named entities and incident types identified by this model could be used a starting point for exploring natural language processing-based incident reporting and learning systems. For example, learning health systems^2^ that help medical professionals retrieve key information from vast corpora of incident reports.

## Methods

**Fig. 1:**
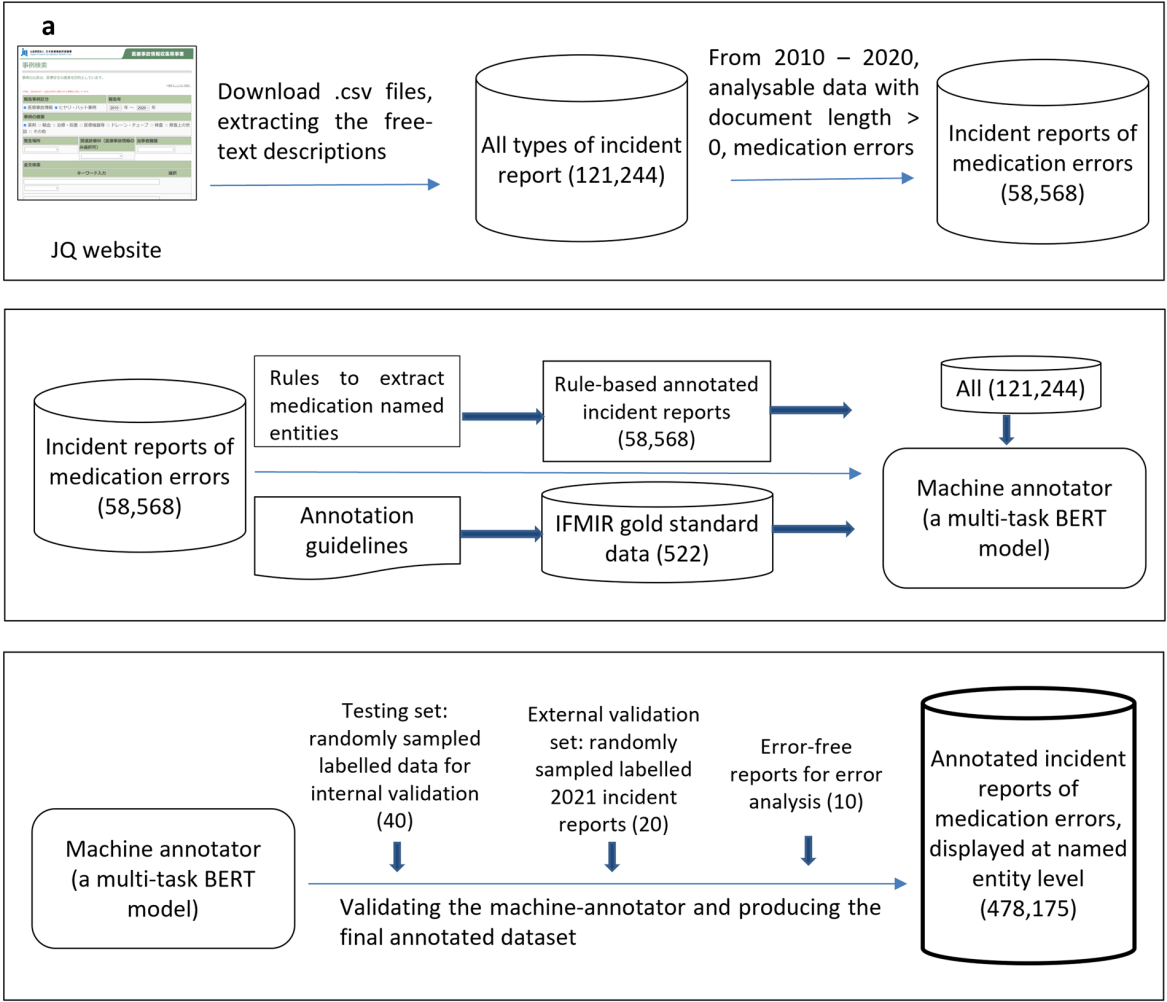
Data creation workflow. (**a**) Building the basic dataset, (**b**) building the machine annotator, which labels named entities, intention/factuality and incident types, and (**c**) technical validation via internal validation, external validation and error analysis.

We present our data creation and validation workflow in Fig. 1 and explain the details below.

### Collecting free-text incident reports of medication errors from JQ

Our source data was obtained from open-access national-wide incident reports collected by JQ’s Project to Collect Medical Near-Miss/Adverse Event Information’ (https://www.med-safe.jp/index.html)^9^. The Project collects incident reports from more than 1,000 medical institutions in Japan. The collected incident reports are made up of structured parts (e.g., forms with drop-down menus) and unstructured parts (e.g., free text in ‘comment’ or ‘note’ sections). For details on the JQ structured reporting items and unstructured reporting guidance, please refer to the supplementary information. In our study, we only used the unstructured narrative reports as the source of free text input. The incident reports in this database are categorised according to their type of error, such as errors relating to medication, blood transfusions, treatment, medical devices, surgical drains, examinations, medical care, etc.

**Fig. 2:**
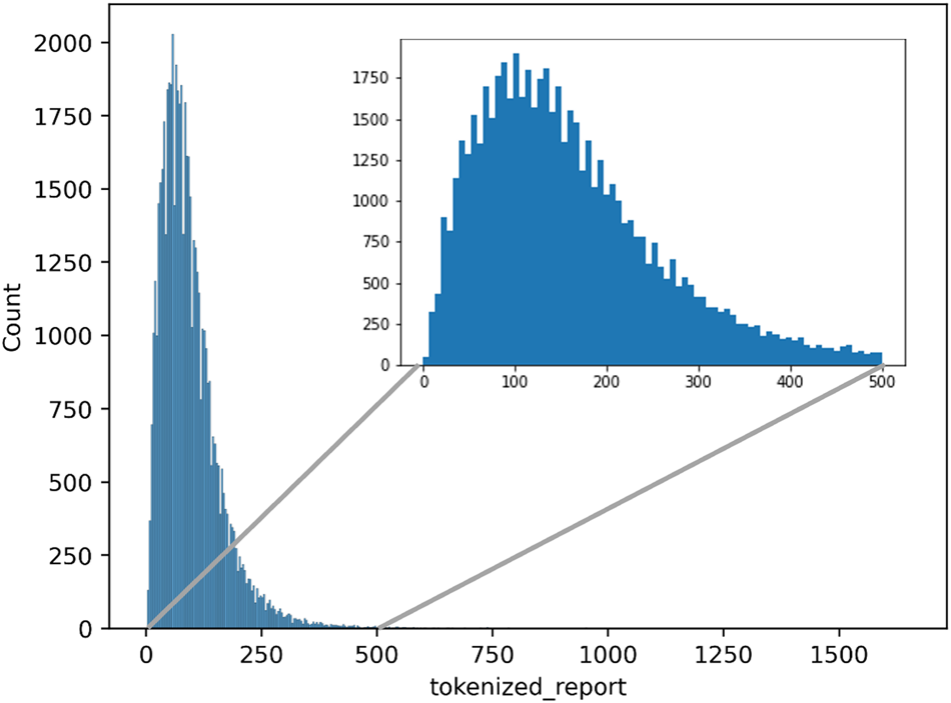
Distribution of incident reports of medication errors (58,658). Y-axis: the number of reports. X-axis: the number of words.

There are 121,244 free-text incident reports in total. Our study focuses on annotating incident reports of medication errors; there are 63,856 medication error reports collected from 2010–2020. We only used 58,568 annotatable free-text incident reports (free-text document length > 0) for annotation, though the entire corpus of 121,244 free-text reports was subsequently used to pre-train the annotation model (as described in Section 3 of the methods). The distribution of report lengths is displayed in Fig. 2. All reports were downloaded in.csv format directly from the JQ website. Subsequently, we randomly selected incident reports from the set of incident reports of medication errors for reference labelling in order to perform validation exercises in the next stage. The technical details of how these annotated datasets were created are included in the supplementary information.

### Annotation scheme

Our annotation scheme is a framework for annotating the information within incident reports in a way that allows medication errors to be extracted systematically. In developing our annotation scheme^19–21^, we referred to reporting guidelines and methods from the World Health Organization’s Minimal Information Model for Patient Safety^13^, the European Medicines Agency’s Good Practice Guide^22^, the NCCMERP index of medication errors^23^ and other relevant studies^24,25^. The annotation scheme and our gold-standard ‘Intention and Factuality Annotated Medical Incident Report’ (IFMIR) dataset have been published previously^20^.

Here, we provide a brief summary of the annotation procedure. First, the drug-related named entities in each report were annotated. Next, each of these named entities was subject to ‘intention/factuality’ analysis, which determined which named entities in the text were *intended* and which named entities were linked to an event that *actually* occurred. Lastly, the type of incident for each report was deduced based on associative rules among the identified annotations. As a reference, our system also produces a relation index, which is shared between named entities belonging to the same event. This index was not included in the technical validation. The main steps are described below:

- **Named entity recognition (NER)**. Named entities are, essentially, the ‘things of interest’ within incident reports – the key units of information that need to be extracted. Named entities that were annotated include ‘drug’, ‘form’, ‘strength’, ‘duration’, ‘timing’, ‘frequency’, ‘date’, ‘dosage’, and ‘route’. Some of these named entities are further separated into subtypes. For example, strength might be described as an amount, e.g., 250 mg, or as a rate, e.g., 250 mg/hr. Note – the current version of the annotation guidelines only covers variables of nouns, proper nouns, compound nouns and numbers.
- **Intention/factuality analysis (I&F)**. All named entities are labelled as ‘intended and actual’ (IA), ‘intended and not actual’ (IN) or ‘not intended and actual’ (NA). The latter two labels indicate the occurrence of a medication error, as a discrepancy exists between what was intended and what actually happened.
- **Incident type**. The incident type, i.e., what kind of medication error occurred, can be determined through an assessment of which named entities were intended and which actually occurred, as well as whether they belong to the primary event. One report might contain more than one incident type. Our pre-defined incidents are as follows: ‘wrong drug’, ‘wrong form’, ‘wrong mode’, ‘wrong strength_amount’, ‘wrong strength_rate’, ‘wrong strength_concentration’, ‘wrong timing’, ‘wrong date’, ‘wrong duration’, ‘wrong frequency’, ‘wrong dosage’ and ‘wrong route’. For errors that fall outside of the above categories, or for free-text inputs that do not contain any error, the incident type is registered as ‘other’.

**Fig. 3:**
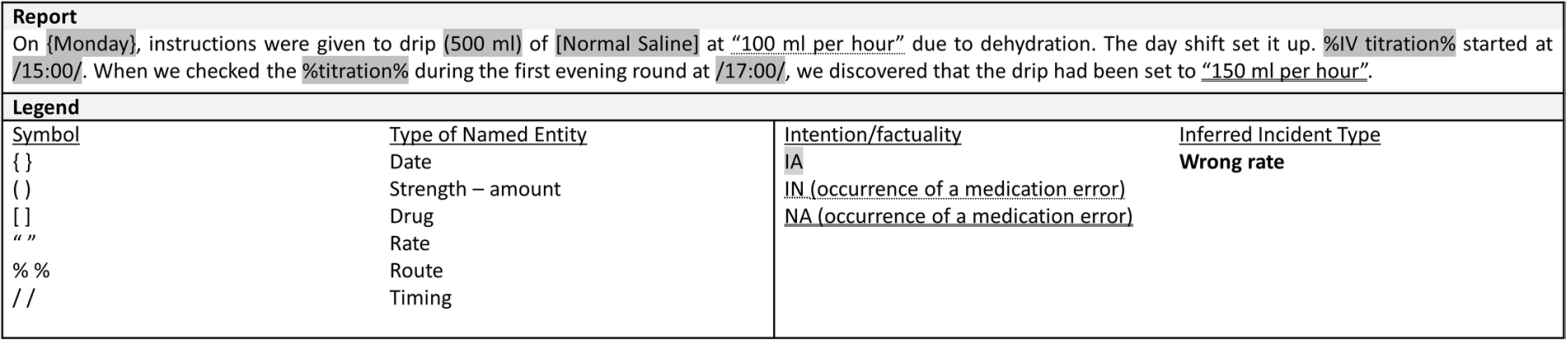
Example of annotation of free text from an incident report. The legend indicates how to distinguish between types of named entity, the results of intention/factuality analysis and relation status. The type of incident inferred by the model is shown on the right.

**Fig. 4:**
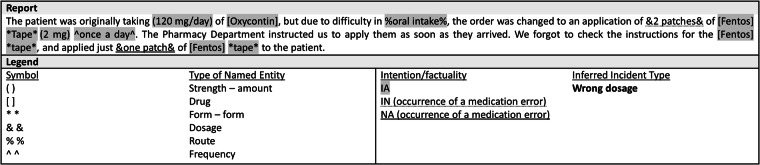
Example of annotation of free text from an incident report. The legend indicates how to distinguish between types of named entity, the results of intention/factuality analysis and relation status. The type of incident inferred by the model is shown on the right.

A detailed explanation of our annotation methodology (June 1, 2023; version 1.0.3) is available online^26^. Figures 3, 4 demonstrate how free-text incident reports are annotated.

### Machine-annotation pipeline

**Fig. 5:**
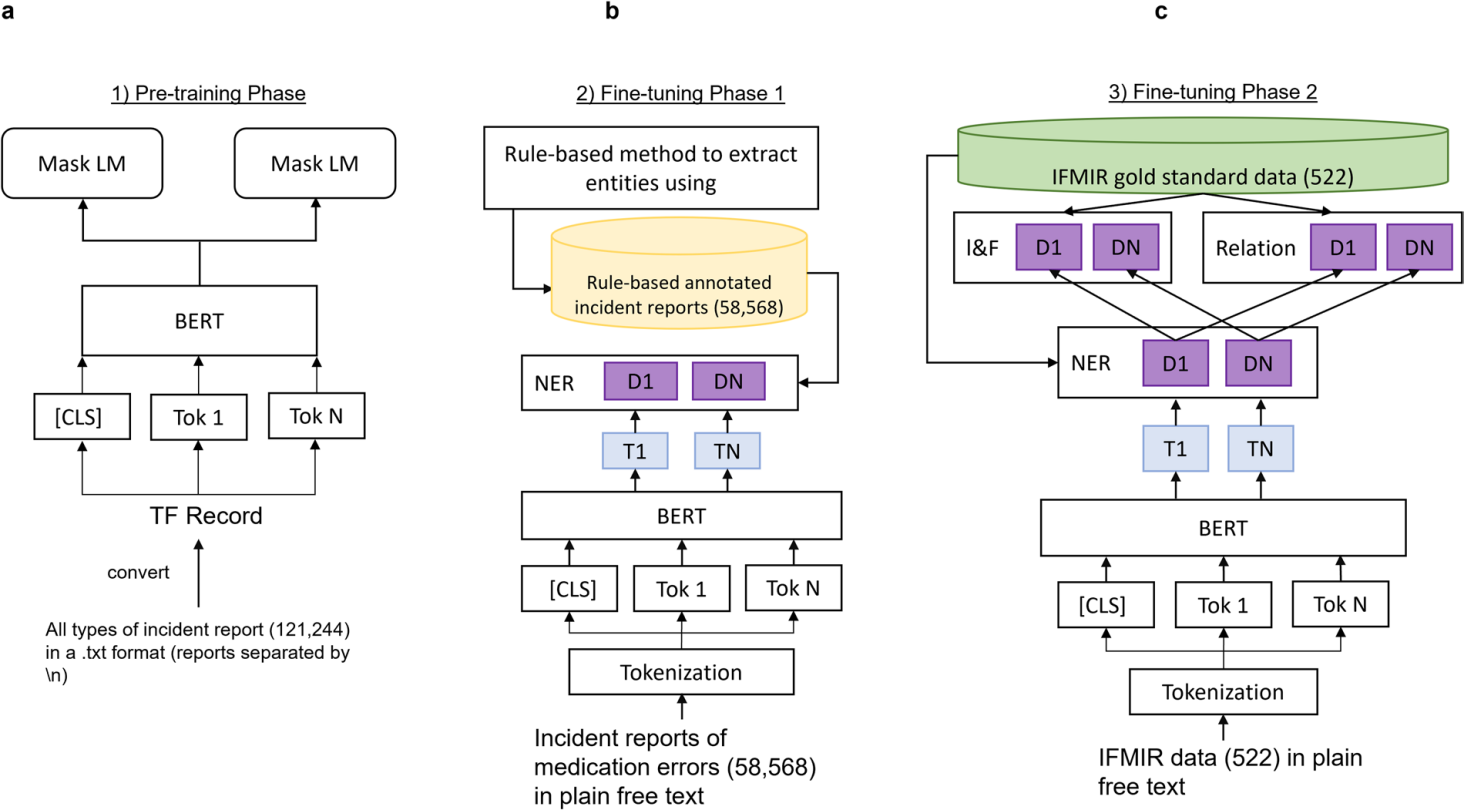
The development of the multi-task BERT machine annotator. (**a**) Pre-training phase, (**b**) fine-tuning phase 1 and (**c**) fine-tuning phase 2.

Using both rule-based and deep-learning approaches, a three-layer multi-task BERT model performed the abovementioned annotation tasks automatically on the extracted free text detailing medication errors, as shown in Fig. 5. The development of the deep-learning pipeline is briefly described below.

#### Pre-training with the full JQ corpus

We adopted a BERT model with the SentencePiece tokenizer pre-trained on Japanese Wikipedia and Twitter corpora (https://github.com/yoheikikuta/bert-Japanese); the BERT model was pre-trained with the JQ incident report corpus of 121,244 free-text documents of all incident types.

#### Fine-tuning with rule-based annotated data

To prepare for the fine-tuning of data in the first phase, we first developed and evaluated an independent rule-based model. We created a list of unique drug names based on the 2022 list of standard drugs published by Japan’s Ministry of Health, Labour and Welfare (https://www.mhlw.go.jp/topics/2021/04/tp20210401-01.html). Furthermore, the lexicons of named entities (including form, mode and route) were obtained from the gold-standard data^20^. For named entities that were often presented in numerical form, such as strength – amount, strength – rate, strength – concentration, frequency, date, dosage, timing and duration, the ‘regular expressions’ (i.e., text sequence/pattern) in which they often appear were recorded and used to identify and extract them. Using the free-text incident reports of medication errors, the rule-based model incorporated morphological segmentation and annotated the targeted named entities according to the abovementioned rules. The resulting annotated incident reports of medication errors were used to fine-tune the pre-trained model at the named entity recognition layer.

#### Fine tuning with gold-standard data

In the second phase of fine tuning, we used the IFMIR gold-standard data (522), to further improve the named entity recognition layer of the BERT model. We treated the intention/factuality task as a problem of named entity-based multiclass classification, and used the known labels from the gold-standard data to fine tune the intention/factuality and relation (reference) layers of the BERT model. 5-fold cross validation was conducted, and we reported the best performing cross-validation result.

## Data Records

**Fig. 6:**
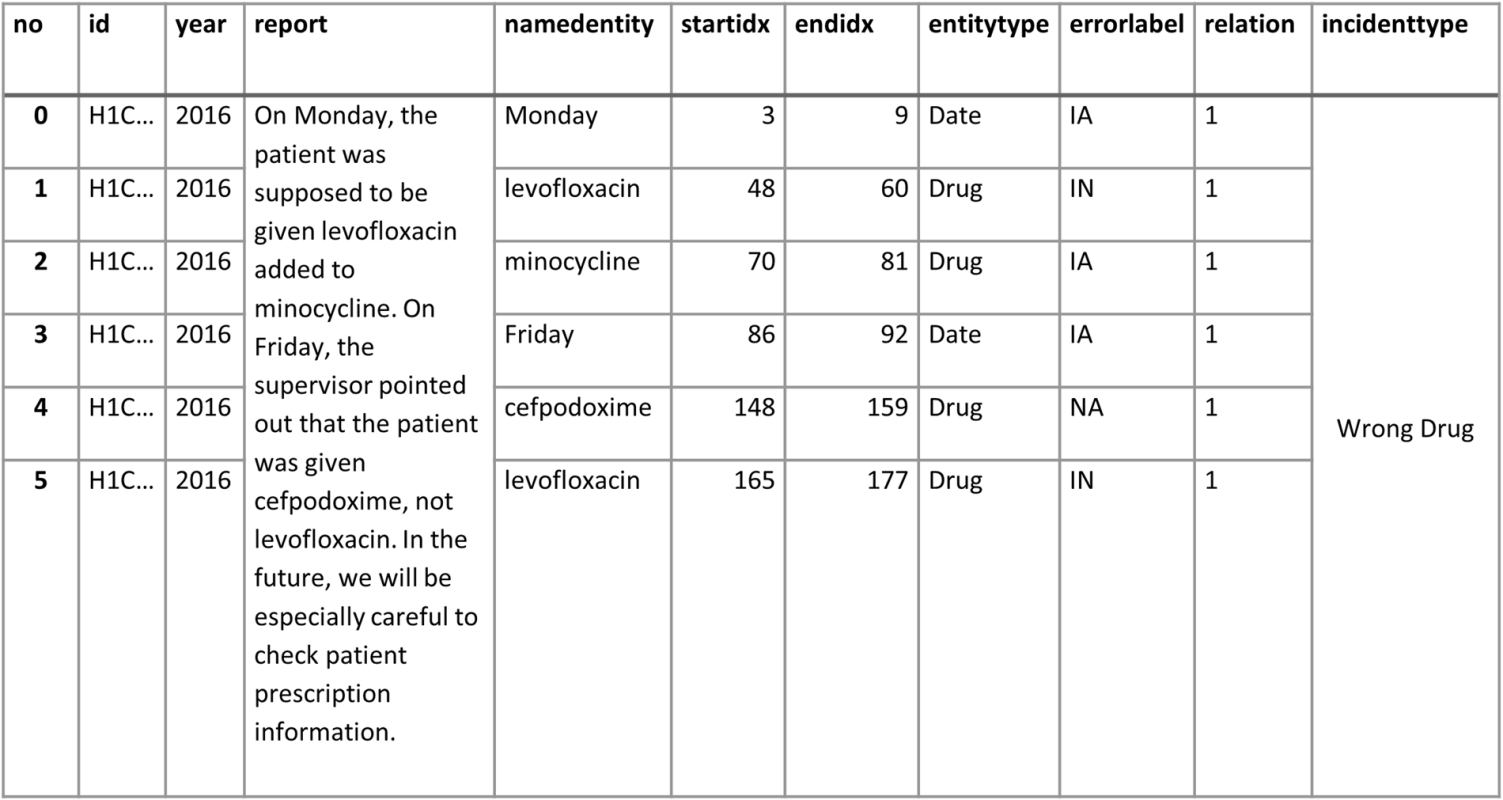
An overview of an annotated incident report.

The final multi-task BERT model was used to annotate the free-text incident reports of medication errors; the deposited dataset at the named-entity level is available on figshare^27^. The corpus is stored as a single.xlsx file. Figure 6 shows the structure of the annotated data, where each row represents a separate named entity. The annotation results for named entity recognition, intention/factuality, and incident type are shown.

We provide a brief explanation of the contents of a typical annotated incident report, as shown in Fig. 6. The first column heading, ‘no’, is the id number of the extracted named entity, ‘id’ refers to the incident report’s ID and ‘year’ refers to the incident reporting year. The heading ‘report’ contains the original free-text report, ‘namedentity’ is the named entity in question, ‘startidx’ is the location index within the report preceding the first character of the named entity and ‘endidx’ is the location index following the final character of the named entity. The column heading ‘entitytype’ refers to the category the named entity belongs to and ‘errorlabel’ is the named entity’s ‘intention/factuality’ status: ‘IA’, ‘IN’ or ‘NA’. The column heading ‘relation’ refers to the index number of the medication event that the named entity belongs to, and ‘incidenttype’ is the inferred incident type described by the report. If multiple types of error occur, this column will display more than one type of incident. In the absence of an error incident, the incident type is indicated as ‘other’. All items in the data that were originally in Japanese text, e.g., ‘report’ or ‘namedentity’, were translated into English using Google Translate (June, 2023); these translated columns are indicated as ‘translated_(heading)’ in the data.

The 58,658 annotated JQ incident reports of medication errors, displayed at the entity level, are available with an English data dictionary at figshare^27^. The AI/NLP pipeline and the readme file for the machine annotator are also available^28^, allowing the multi-task BERT model to be applied towards annotating other free-text incident reports of medication errors.

Furthermore, we share the manually reviewed datasets used for technical validation on figshare^29^. These are the randomly sampled labelled incident reports from 2010–2020 (n = 40) for testing/internal validation, randomly sampled labelled incident reports from 2021 (n = 20) for external validation, and error-free reports (n = 10) for error analysis. The procedure for preparing these labelled datasets for technical validation is described in the supplementary information.

## Technical Validation

**Fig. 7:**
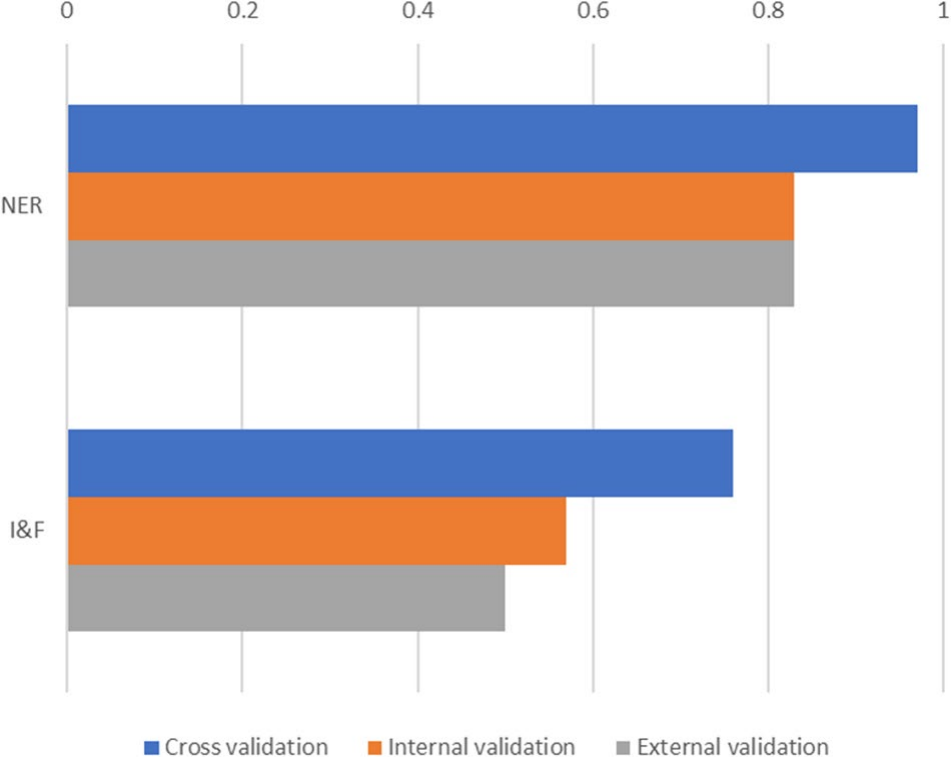
Technical validation summary. Reported F-1 scores for the cross validation, internal validation and external validation exercises.

All datasets used for technical validation can be found on figshare^29^. The supplementary information provides all details of technical validation, which comprised cross-validation, internal validation, external validation and error-analysis. We briefly summarise the main results of the cross-validation, internal and external validations in Fig. 7. 5-fold cross validation was conducted to search for model parameters using the IMFIR gold standard data. In the internal validation phrase, we randomly selected 40 incident reports from the 58,568 incident reports of medication errors from 2010–2020 and manually reviewed the reference labels of the selected incident reports. In total, this internal validation dataset contains 263 identified named entities. Since this set of validation data was within the produced annotated dataset, this validation exercise revealed the extent to which the labels are correctly identified within the deposited dataset. Furthermore, we conducted external validation using 20 external reports extracted from 2021 and this set of data contains 110 named entities identified by manual review. The performance evaluation metrics of precision, recall and F1-score are reported in these exercises. The best performing F1-scores at cross-validation for NER and I&F were 97%, and 76%, respectively. In the internal validation exercise, the macro-average of the F1-score across all named entities was 83% and 57% for I&F tasks. In the external validation exercise, these same two metrics were 83% and 50%, respectively. In error analysis, the machine annotator achieved 90% accuracy (false positive rate of 10%) in correctly identifying reports without error.

## Usage Notes

This deposited dataset characterises medication incident reports in a structured and machine analysable format. In this way, the dataset serve as a library of similar incidents, connecting users with a wide variety of relevant past cases. Search queries can draw upon combinations of annotations for named entities, intention/factuality and incident types, facilitating the efficient retrieval of similar or relevant cases, particularly high-risk scenarios. For instance, using the identified drug named entity, one can retrieve past incidents of multiple-drug consumption situations, such as polypharmacy and look-alike-sound-alike medications. Furthermore, among the incidents involving incorrect dosages, one can further pinpoint opioid-based drugs to better understand instances of opioid misuse. Furthermore, the machine annotator is capable of on-demand annotation of other free-text incident reports of medication errors in the Japanese language, such as new JQ incident reports, or those collected by individual hospitals.

Digital health system designers can use the machine annotator and the annotated corpus for developing incident report systems, providing function to automated knowledge extraction and retrieval of relevant past cases. We previously demonstrated a proof-of-concept system online (https://www.aiforpatientsafety.com/). The machine annotator can also be further fine tuned and repurposed for other applications related to medication errors, e.g., the free text found in electronic health records.

Incident reports are often proprietary data and are not shared publicly. This open, annotated dataset, generated using ‘real life’ incident reports, has been translated into English for users in other countries. This serves as a valuable and unique resource for systematic studies of medication errors around the world. The workflow and annotation guidelines were designed to be usable in other languages and have been experimentally tested using English incident reports. This large dataset of real-world reports can serve as standard annotated data for natural language processing challenges, examples of which include drug–drug interaction challenges^30,31^, medication errors/adverse drug challenges^32^ and n2c2 challenges^33^.

Annotated data associated with this Data Descriptor are available at figshare^27,29^ and are released under CC-BY 4.0 to maximise reuse and further study. The original data used to create these reports were collected as part of JQ’s ‘Project to Collect Medical Near-Miss/Adverse Event Information’. When using this deposited dataset, one should also cite the original data source from JQ (https://www.med-safe.jp/index.html).

As described in the section on technical validation, we used samples of randomly selected reports that were manually annotated to validate our model. Despite our best efforts to make the delivered datasets as accurate as possible, some errors might remain due to the high variability in content across reports and the inability to thoroughly validate every label. Please email the corresponding author of this paper to report any errors or provide other comments.

### Supplementary information

Supplementary Information

## Data Availability

The machine annotator code is written in Python 3.9, using libraries for pandas, NumPy, multiprocessing, tqdm, transformers, SentencePiece, neologdn, etc. and can be operated on Google Colab or a local integrated development environment. The annotation guidelines^26^, deposited dataset^27^ and machine annotator^28^ are available at figshare. The manually reviewed and machine-annotated datasets used for technical validation are also available at figshare^29^.
