# Supplementary material for: A large dataset of annotated incident reports on medication errors
Source: Sci Data. 2024 Feb 29;11:260. doi: 10.1038/s41597-024-03036-2 (PMC10904777; doi:10.1038/s41597-024-03036-2)
Supplement: Supplementary file 1 — Supplementary Information [file 41597_2024_3036_MOESM1_ESM.docx]

Table of Contents

# 1. Reporting Form Structure of ‘Project to Collect Medical Near-Miss/Adverse Event Information’2

# 2. Technical validation4

## 2.1 Preparation of validation datasets4

## 2.2 Summary of technical validation datasets5

### Table S1 6 Figure S1 9

## 2.3 Procedure to generate receiver operating characteristic (ROC) curves and precision-recall (PR) curves 10

## 2.4 Cross-validation (IFMIR gold standard data; n = 522)11

### Table S2 11

## 2.5 Internal Validation (randomly sampled labelled incident reports from 2010 – 2020; n = 40)11

### Table S3 12 Figure S2 14

### Figure S3 15

### Figure S4 15

### Figure S5 16

### Figure S6 17

### Figure S7 17

### Figure S8 18

### Figure S9 18

### Figure S10 19

## 2.6 External Validation (randomly sampled labelled incident reports from 2021; n =20) 19

### Table S4 20

### Figure S11 22

### Figure S12 23

### Figure S13 24

### Figure S14 25

### Figure S15 26

### Figure S16 26

### Figure S17 27

### Figure S18 27

### Figure S19 28

## 2.7 Error Analysis (error-free reports; n = 10) 28

# 3. References29

1. Reporting Form Structure of ‘Project to Collect Medical Near-Miss/Adverse Event Information’

Below is an English translation of the table of contents for the ‘Guidelines on how to fill out medical incident reports’ (<https://www.med-safe.jp/pdf/accident_essentials.pdf>) for the ‘Project to Collect Medical Near-Miss/Adverse Event Information’^1^. This table of contents provides an overview of what is reported when Japanese healthcare professionals complete medical incident reports. Such reports were used in this study.

Structured parts (e.g., forms with drop-down menus)

**(1) Summary of the accident**

(1) Year of incident

(2) Month of incident

(3) Day of the incident

(4) Day of the week

(5) Time of the incident

(6) Whether medical care was provided

(7) Extent of the accident

(8) Location of the accident

(9) Summary

(10) Cases requiring special reporting

(11) Related departments

**(2) Information on patients**

(1) Number of patients

(2) Patient classification

(3) Name of disease

(4) Patient's condition before the last visit

**(3) Information on the parties concerned**

(1) Parties concerned

(2) Occupation of the parties concerned

(3) Professional history of the party

(4) Period of professional assignments

(5) Number of duty/night shifts in the last 1 week before the incident

(6) Type of work

(7) Working hours in the last 1 week

(8) Discoverer

**(4) Information on parties other than the parties**

(1) Occupations of parties other than the parties concerned

**5) Information on the scene and content of the incident**

(1) Type of incident

(2) Scene

(3) Details of the incident

(4) Medicine/drugs

(5) Medical equipment, etc.

(6) Medical materials

Unstructured parts (e.g., free text in ‘comment’ or ‘note’ sections)

**(6) Information on the nature of the accident (by narrative text)**

(1) Purpose of the medical treatment performed

(2) Details of the accident

(3) Causes of the accident

(4) Outline of background factors of the accident

(5) Whether an accident investigation committee has been established

(6) Improvement measures

1. **Technical Validation**

A complete technical validation was conducted to evaluate the validity of the data and the extent to which the machine annotator can be applied to free-text reports not used in the model development phase. Figure 1, included in the main manuscript, summarises the validation phase**.** Technical validation consists of cross-validation during model training, internal validation, external validation and error analysis. This report summarises the data creation, procedures and results of each of these exercises.

2.1 Preparation of validation datasets

In this section, we summarise the procedure for preparing the labelled datasets for each phase of the technical validation, see below. The annotation was done in accordance with the annotation guidelines for incident reports of medication errors^2^.

- **Training/Cross-validation (IFMIR gold standard data; n = 522)**: Details on how the IFMIR gold standard data were created have already been published^3^. The original annotated data (in .ann format) can be found on GitHub^4^. This set of labelled data is regarded as the gold standard, with a reported F-1 score of 0.960 for inter-annotator agreement.
- **Testing/Internal Validation (randomly sampled labelled incident reports from 2010** **– 2020; n = 40):** We took 40 random samples from the incident reports of medication errors (58,568) from 2010 – 2020 and labelled the free-text reports according to the annotation guidelines. In total, this internal validation dataset contains 263 identified named entities. Randomization was done to ensure that the samples were a representative subset of the incident reports, and the validation exercise was done in a generalised, non-biased way. We named this annotated dataset ‘randomly sampled labelled data (40)’. In the case of uncertain labelling/annotation, another senior annotator reviewed the report and made final judgement.
- **External Validation (randomly sampled labelled incident reports from 2021; n = 20)**: We took 20 random samples from the incident reports of medication errors from 2021, provided by the Japan Council for Quality Health Care (JQ)^5^, and labelled the free-text reports according to the annotation guidelines. The reports were retrieved in mid-May, 2023. There was a total of 5,256 incident reports on the JQ website^5^, 415 of which were adverse events and 4,841 near misses. We registered every report using the traceable incident ID produced by JQ. Among the 20 annotated reports, two were classified as adverse events and 18 were near misses. This set of data contains 110 named entities, identified by manual review. We named this annotated dataset ‘randomly sampled labelled data (20)’. In the case of uncertain labelling/annotation, another senior annotator reviewed the report and made final judgement.
- **Error analysis (error-free reports; n = 10)**: We performed a series of error analyses to examine the validity and robustness of the machine annotator. The goal was to investigate the machine annotator’s ability to correctly recognise free-text inputs that do not contain any errors. To do so, we created 10 synthetic, error-free narrative texts that fell under one of two categories, described below. We named this dataset ‘error-free reports (10)’. The two types of synthetic text were as follows:
- Error-free, medication-related free text (n = 5): To create these texts, we randomly selected five reports from the dataset of 58,568 incident reports of medication errors. Based on the content of these reports, we wrote five error-free reports that involved drug concepts and numbers similar to the reports randomly selected.
- Error-free and non-medication-related free text (n = 5): These were randomly extracted from an open source, specifically the webpage ‘Information on how to apply for the Tokyo News’ [Tōkyō shinbun o mōshikomi no go annai]^6^.

2.2 Summary of technical validation datasets

Here we present the summary statistics of the three free-text datasets used in the study (Table S1) and show the graphical distribution of the datasets used in this study (Figure S1 a–e).

**Table S1.** Descriptive statistics of different datasets used in the study.

|  | **Incident reports of medication errors (58,568)** | **IFMIR gold standard annotated data (522)** | **Randomly sampled labeled data from 2010-2020 (40)** | **Randomly sampled labelled incident reports from 2021 (20)** | **Error-free reports (10)** |
| --- | --- | --- | --- | --- | --- |
| **Descriptive Statistics** |  |  |  |  |  |
| N of reports | 58,389 | 517 | 40 | 20 | 10 |
| N of identified entities | 478,176 | 3,125 | 263 | 110 | 8 |
| N of tokens/ Total length | 5,660,104 | 22,940 | 2,665 | 1,544 | 273 |
| Maximum length of reports | 1,651 | 81 | 181 | 235 | 53 |
| Minimum length of reports | 3 | 17 | 14 | 10 | 13 |
| Mean (SD) length of reports | 96.95 (70.62) | 44.08 (15.08) | 66.63 (37.62) | 77.20 (60.02) | 27.30 (11.59) |
| Median (IQR) length of reports | 82 (73) | 43 (25.0) | 63.5 (43.25) | 60 (50.75) | 25 (5.00) |
| Annotation quality | Predicted^#^, Good quality - as refer to the Technical Validation | Highest^$^ | High^%^ | High^%^ | High^%^ |
| Entities per reports | 8.19 | 5.70 | 6.60 | 5.5 | 0.8 |
| **Entity Type (N, percentage)** |  |  |  |  |  |
| Drug | 168,633 (35.27%) | 1,093 (34.98%) | 93 (35.36%) | 48 (13.83%) | 4 (50%) |
| Date | 40,617 (8.49%) | 171 (5.47%) | 27 (10.27%) | 9 (2.59%） | 1 (12.5%) |
| Route | 36,496 (7.63%) | 139 (4.45%) | 22 (8.37%) | 19 5.48%） | 0 (0) |
| Dosage | 22,731 (4.75%) | 228 (7.30%) | 17 (6.46%) | 5 (1.44%） | 1 (12.5%) |
| Duration | 15,127 (3.16%) | 93 (2.98%) | 10 (3.80%) | 3 (0.86%） | 0 |
| Timing | 90,986 (19.0%) | 313 (10.02%) | 50 (19.01%) | 11 (3.17%） | 1 (12.5%) |
| Strength – amount | 59,493 (12.4%) | 645 (20.64%) | 21 (7.98%) | 11 (3.17%） | 1 (12.5%) |
| Strength – rate | 8,084 (1.69%) | 71 (2.27%) | 3 (1.14%) | 0 (0) | 0 |
| Strength – concentration | 4,041 (0.85%) | 27 (0.86%) | 5 (1.90%) | 0 (0) | 0 |
| Form – form | 22,859 (4.78%) | 248 (7.94%) | 10 (3.80%) | 3 (0.86%) | 0 |
| Frequency | 7289 (1.52%) | 80 (2.56%) | 5 (1.90%) | 1 (0.29%) | 0 |
| Form_Mode | 1,806 (0.38%) | 17 (0.54%) | 0 (0) | 0 (0) | 0 |
| **I&F distribution (N, percentage)** |  |  |  |  |  |
| IA | 348,088 (72.79%) | 1,730 (55.36%) | 168 (63.88%) | 71 (64.55%) | 8 (100%) |
| IN | 57,939 (12.11%) | 602 (19.26%) | 63 (23.95%) | 17 (15.45%) | 0 |
| NA | 71,909 (15.04%) | 630 (20.16%) | 32 (12.17%) | 22 (20%) | 0 |

^#^ The predicted labels were generated by the machine annotator based on the BERT model fine-tuned with the IFMIR gold standard dataset.

^$^ Gold-standard labels were created by an external linguistic company and evaluated based on measures agreed upon by multiple evaluators^3^.

^%^ Near gold-standard quality labels were developed by careful manual review and reconfirmed by a senior annotator.

| 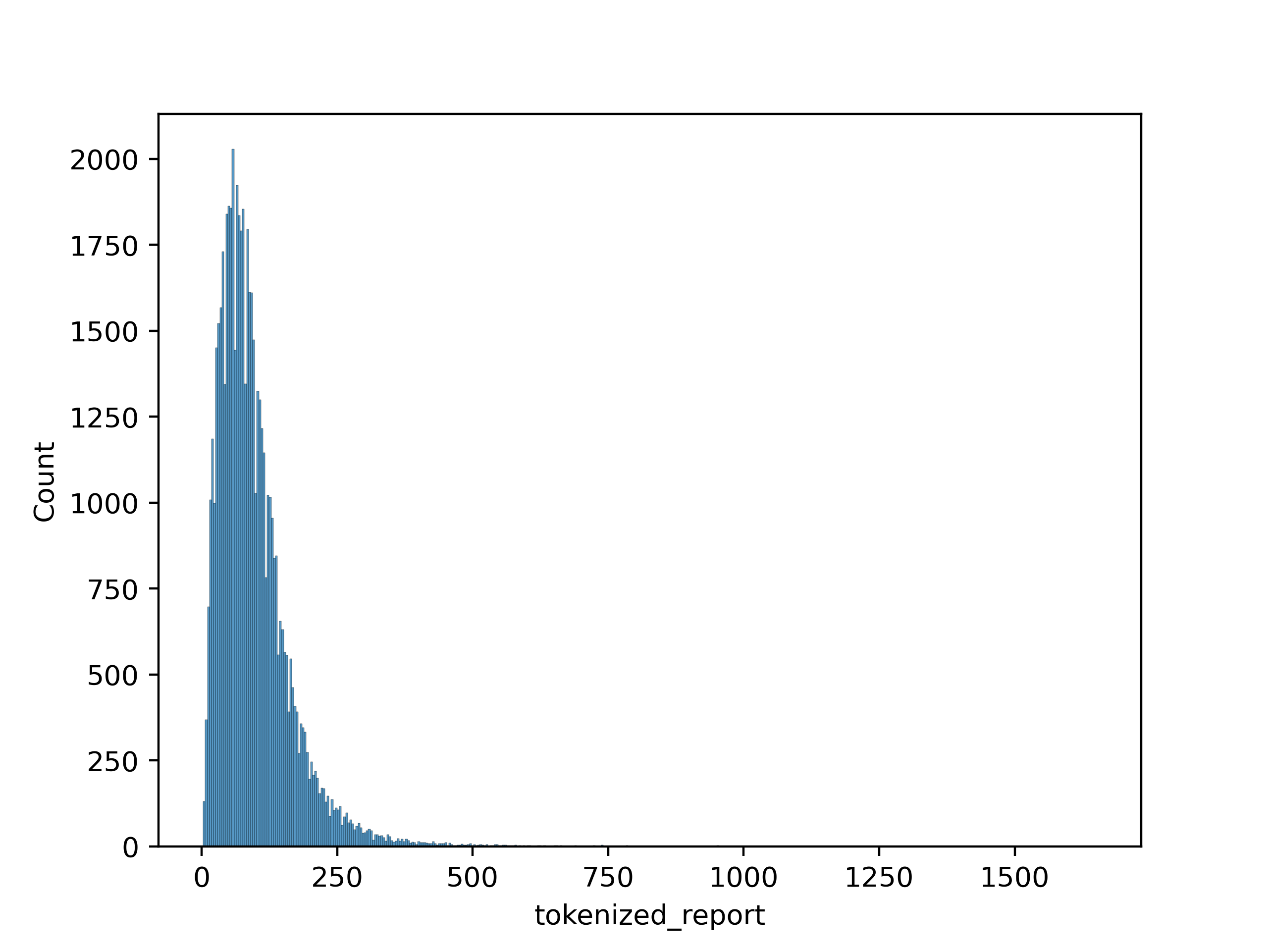 | 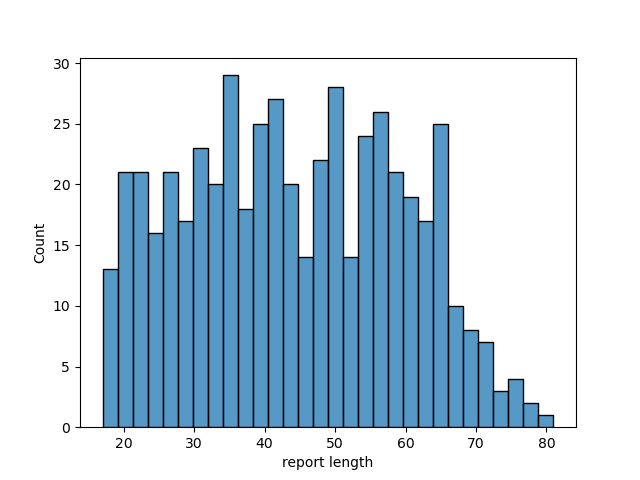 |
| --- | --- |
| **a** | **b** |
| 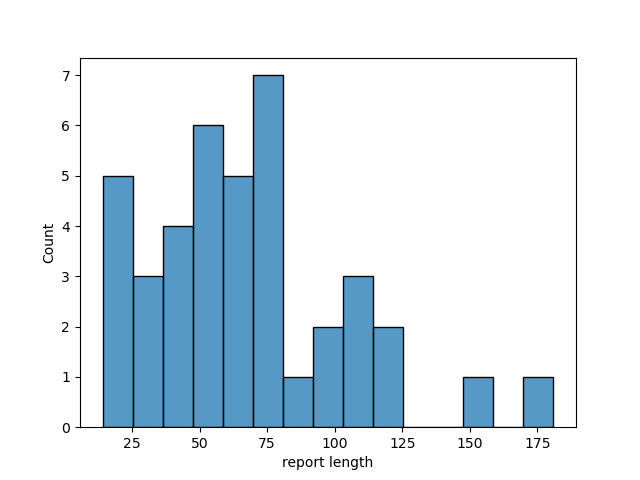 | 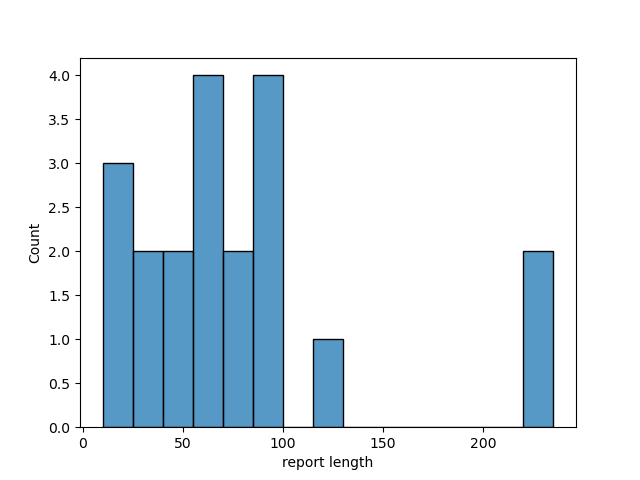 |
| **c** | **d** |
| 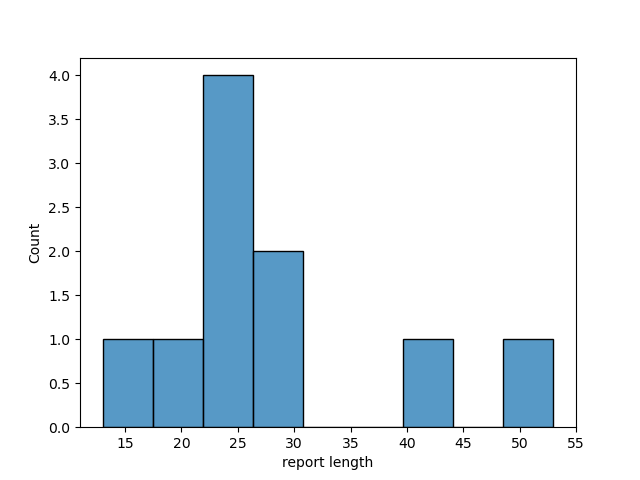 |  |
| **e** |  |

**Figure S1.** Distributions of incident reports of medication errors. **a)** Incident reports of medication errors (58,568), **b)** IFMIR gold-standard annotated data (522), **c)** randomly sampled labelled data from 2010 – 2020 (40), **d)** randomly sampled labelled incident reports from 2021 (20), and **e)** error-free reports (10).

Using the abovementioned datasets, we performed strict model evaluation using the Python framework seqeval^7^ to evaluate the performance of the machine annotator, in terms of Precision, Recall and F1-score. We present the results of evaluation for overall annotation tasks and performance at the more detailed named entity/class level. For named entity/class level performance, we conducted evaluation with the ‘O’ object class (i.e., non-named entity) included as it relates to the model’s ability to distinguish objects from different named entity classes and non-named entity classes. In terms of evaluation for intention/factuality (I&F), as this prediction is solely based on whether concepts are correctly identified as named entities, the evaluation scheme should not involve the object class ‘O-1’. The above is deemed to be a fair evaluation for a problem of named entity classification and attribute identification. It should also be noted that there is no NER system designed for medication error-related incident reports in the existing literature, and that our system is applied to the Japanese language, so we are unable to compare the model’s performance with any baseline.

2.3 Procedure to generate receiver operating characteristic (ROC) curves and precision-recall (PR) curves

For the internal validation (using randomly sampled labelled incident reports from 2010 – 2020; n = 40) and external validation (using randomly sampled labelled incident reports from 2021; n = 20), we visualised the data using the receiver operating characteristic (ROC) curves and precision-recall (PR) curves, and calculated their corresponding values for area under the curve (AUC) and average precision (AP). As the creation of these metrics is not trivial for the NER problem (which involves multiple tokens’ multiclass classification in a sequence), we explain the procedure below:

For the NER problem, based on the multiclass-ROC procedure provided by scikit-learn 1.3.2 (<https://scikit-learn.org/stable/auto_examples/model_selection/plot_roc.html>), we further binarized the token-based NER outputs using the one-vs-the-rest (OvR) scheme, which compares each class against all the others. NEs were identified based on each token’s B-I-O status, which indicates whether the token is found at the beginning (B) or inside (I) of an NE, or outside of any NEs (O). It was thus possible to further break these down into multiple token-level binary classification problems. As shown in Table S1, the testing/internal validation dataset (n = 40) covers 11 uniquely identified NEs and as a result, there are 24 classes in total (i.e., 11 B-type NEs, 11 I-type NEs, 1 O-type NE and 1 PAD-type NE). For the external validation dataset (n = 20), there are 19 classes (including all B-I-O statuses and PAD).

The BERT machine annotator produces vector outputs for each token. For each token, after SoftMax layer transformation, we could obtain a set of probabilities (the sum of which = 1) across all the possible predicted classes. Having these, we could count data instances across every token for each class and rank these class-based data samples according to its order of magnitude. We first removed all the irrelevant “PAD” tokens* and used each (one) class to compare with the rest of the classes (-vs-the-Rest) to evaluate the NER performance**. For instance, we calculate the probability of there being a match for drug B (p) and there being no match (1-p), thereby determining the true positive rate (TPR) (i.e., sensitivity) and false positive rate (FPR) (i.e., 1-specificity).

*PAD is a data-length batching technique in BERT and is irrelevant to the prediction

**During the standard sequence-evaluation procedure, conducted by Python seqeval, NE-based metrics in terms of precision, recall and F-score, as presented above, were produced by incorporating post-processing rules for sequence (as NER often involves multiple tokens in sequence) to evaluate whether NEs were correctly identified. Therefore, the binary-classification regime, which only considers the class identification of individual token, could only provide partial indication of NER performance. Therefore, the precision, recall and F-score are regarded as the standard assessment than AUC and PR curves.

Similarly, we used the multi-class classification settings of precision-recall curve provided by scikit-learn (<https://scikit-learn.org/stable/auto_examples/model_selection/plot_precision_recall.html>) to produce the PR curves for the NER problem. For the I&F problem (a typical NER-based multi-class classification problem), we simply evaluated the three classes of IA, IN and NA. Micro-averaging strategy, which is preferable under imbalanced class properties (as revealed in Table S1), were used to average all ROC curves and PR curves at each threshold.

2.4 Cross-validation (using IFMIR gold standard data; n = 522)

Using the IFMIR gold standard data training data, 5-fold cross validation was performed at the second phase of model fine-turning. The best macro average across 5-fold validation for named-entity recognition (NER) and I&F were 0.97 and 0.76, respectively. Here we report the overall cross validation results in detail.

**Table S2.** Model performance at k-fold cross-validation (k = 5) stage using IFMIR gold standard dataset. The best macro-average of precision, recall and F1-score across k validation folds for each overall task are reported.

| The best macro-averaged scores across k-fold (k=5) | Precision | Recall | F1-score |
| --- | --- | --- | --- |
| NER | 0.97 | 0.97 | 0.97 |
| I&F | 0.75 | 0.76 | 0.76 |

NER: named entity recognition; I&F: intention/factuality; IA: intended and actual; IN: intended and not actual; NA: not intended and not actual. Note: the statistics were captured in a token-level manner.

2.5 Internal Validation (using randomly sampled labelled incident reports from 2010 – 2020; n = 40)

Internal validation aims to examine the extent to which the model performs well within the original dataset. Since this set of validation data was within the produced annotated dataset, this validation exercise also revealed the extent to which labels are correctly identified within the shared dataset. In the internal validation exercise, the macro-average for the F1-score across all named entities was 83%; for I&F tasks it was 57% (see Table S3). The overall accuracy for predicting incident type (as a multi-label problem) was 60%. We also summarise the internal validation results (in terms of overall task performance, performance at the named entity level and I&F class prediction) in Figure S2.

For NER tasks, AUC values across different OvR classifications ranged from 0.85 to 1, as shown in Figure S3 (a & b). Figure S4 presents the micro-average ROC curve with AUC = 1. Figure S5 (a & b) presents the PR curves for all OvR classifications, demonstrating AP values from 0.74 to 1, and Figure S6 shows the micro-averaged PR curve with an AP value of 0.97.

Figures S7 and S8 present the ROC curves for the I&F tasks and their micro-average ROC curve, demonstrating a micro-averaged AP of 0.83. Figures S9 and S10 show the PR curves by class (IA, IN, and NA) and their micro-average curve, demonstrating a micro-average AP value of 0.75.

**Table S3.** Performance evaluation of the internal validation. The precision, recall and F1-score for each overall task and subtask are reported. Overall I&F accuracy was 0.66; class-specific scores for I&F are below.

| Marco-averaged | Precision | Recall | F1-score |
| --- | --- | --- | --- |
| NER | 0.81 | 0.84 | 0.83 |
| I&F | 0.57 | 0.57 | 0.57 |

|  | **Precision** | **Recall** | **F1-score** |
| --- | --- | --- | --- |
| **Date** | 0.80 | 0.74 | 0.77 |
| **Dosage** | 0.87 | 0.76 | 0.81 |
| **Drug** | 0.82 | 0.76 | 0.79 |
| **Duration** | 0.82 | 0.90 | 0.86 |
| **Form – form** | 0.91 | 1.00 | 0.95 |
| **Frequency** | 0.80 | 0.80 | 0.80 |
| **Route** | 0.69 | 0.82 | 0.75 |
| **Strength – amount** | 0.74 | 0.81 | 0.77 |
| **Strength – concentration** | 0.67 | 0.80 | 0.73 |
| **Strength – rate** | 1.00 | 1.00 | 1.00 |
| **Timing** | 0.84 | 0.86 | 0.85 |

|  | Precision | Recall | F1 |
| --- | --- | --- | --- |
| IA | 0.82 | 0.78 | 0.80 |
| IN | 0.49 | 0.46 | 0.47 |
| NA | 0.40 | 0.48 | 0.43 |

NER: named entity recognition; I&F: intention/factuality; IA: intended and actual; IN: intended and not actual; NA: not intended and not actual. Macro-average scores are presented. Note: the statistics were captured in a named-entity-level manner.

**Figure S2.** Internal validation results. **a)** Overall NER and I&F results, **b)** evaluation at the named-entity level and **c)** I&F evaluation.


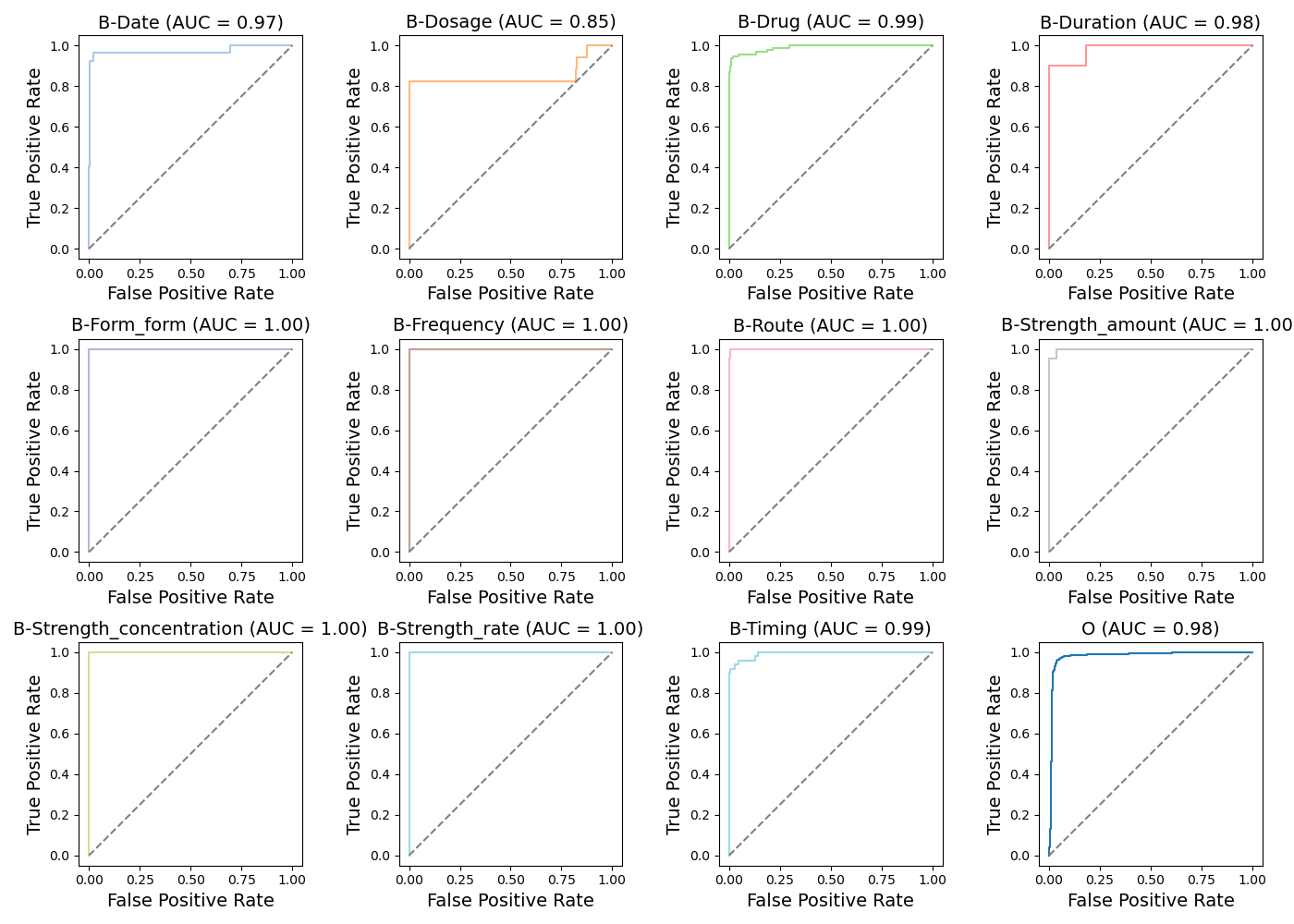


**Figure S3(a).** Internal validation visual results (NER) – 12 ROC curves for 11 NEs (as B) and 1 outside (as O) and their corresponding AUC values.


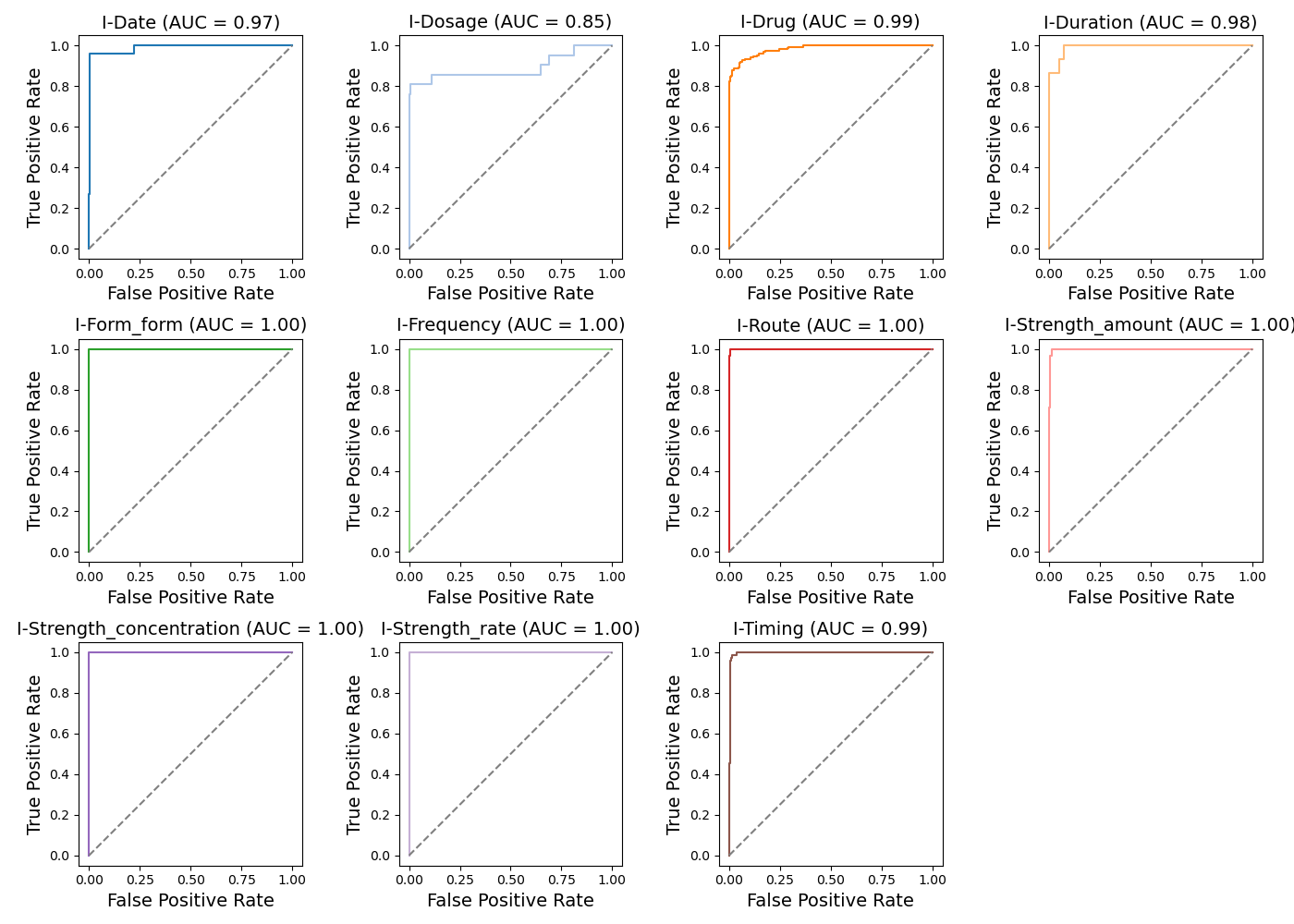


**Figure S3(b).** Internal validation visual results (NER) – 11 ROC curves for 11 NEs (as I) and their corresponding AUC values.


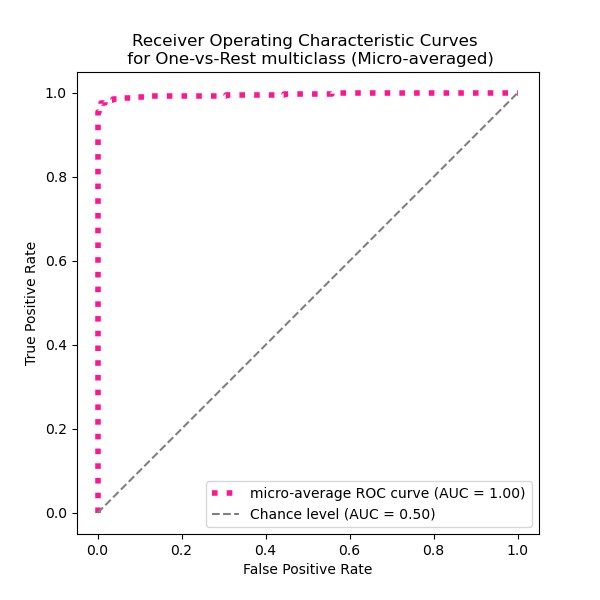


**Figure S4.** Internal validation visual results (NER) – micro-average ROC curve and their corresponding AUC values.


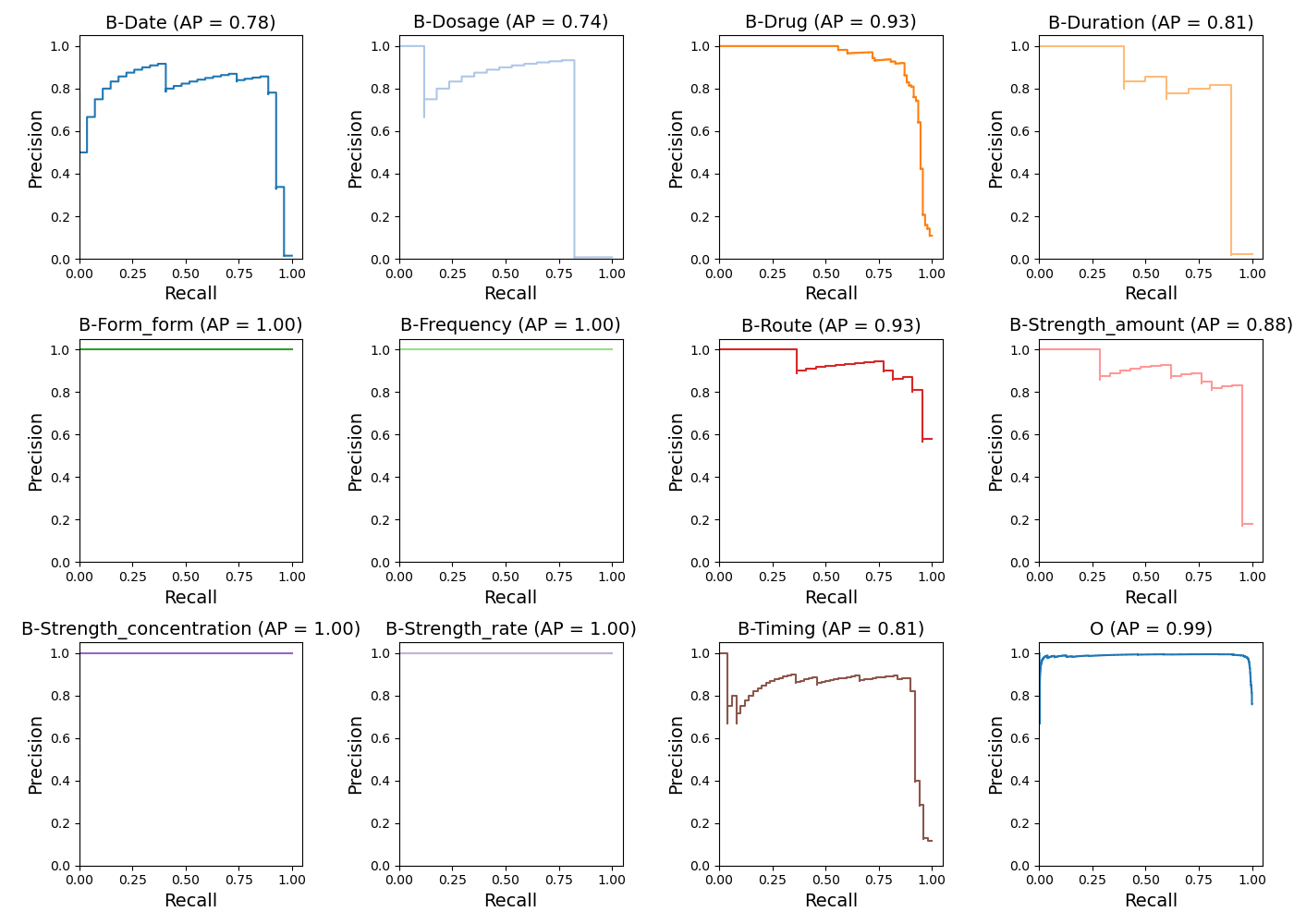


**Figure S5(a).** Internal validation visual results (NER) – 12 PR curves for 11 NEs (as B) and 1 outside (as O) and their corresponding AP values.


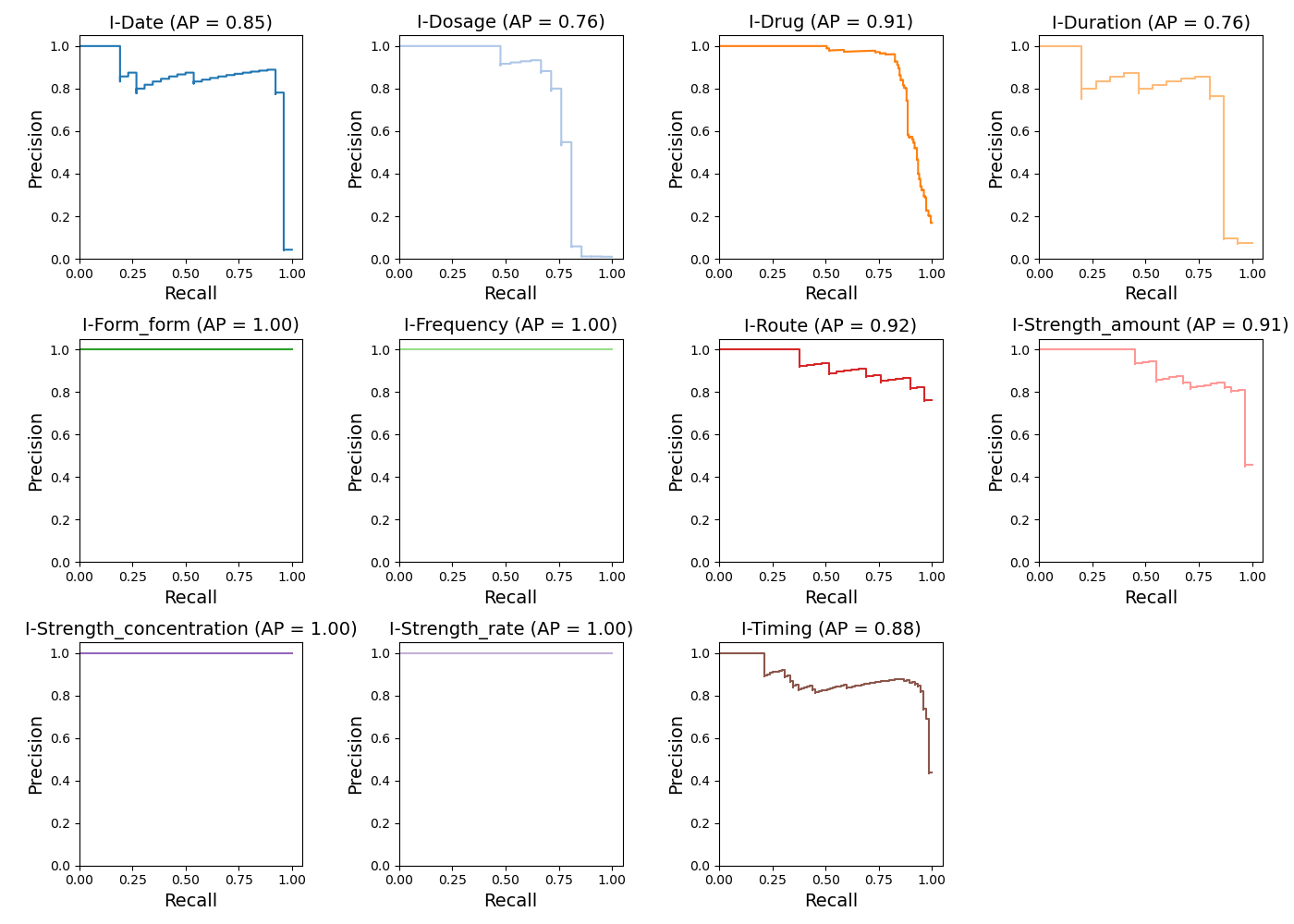


**Figure S5(b).** Internal validation visual results (NER) – 11 PR curves for 11 NEs (as I) and their corresponding AP values.


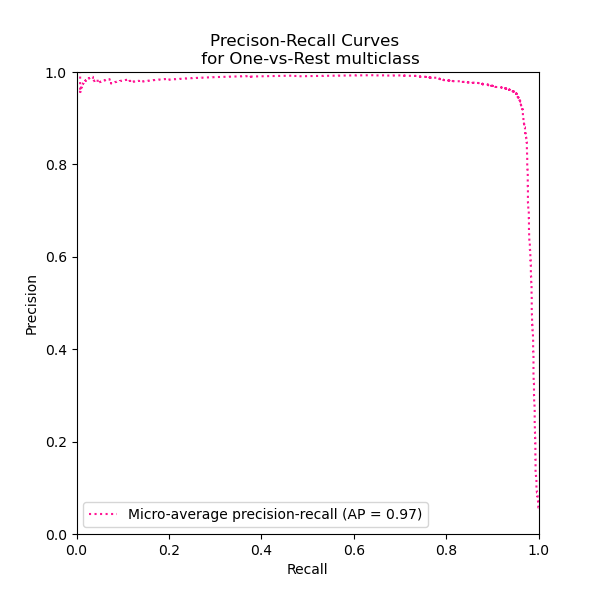


**Figure S6.** Internal validation visual results (NER) – micro-average PR curve and their corresponding AP values.

**
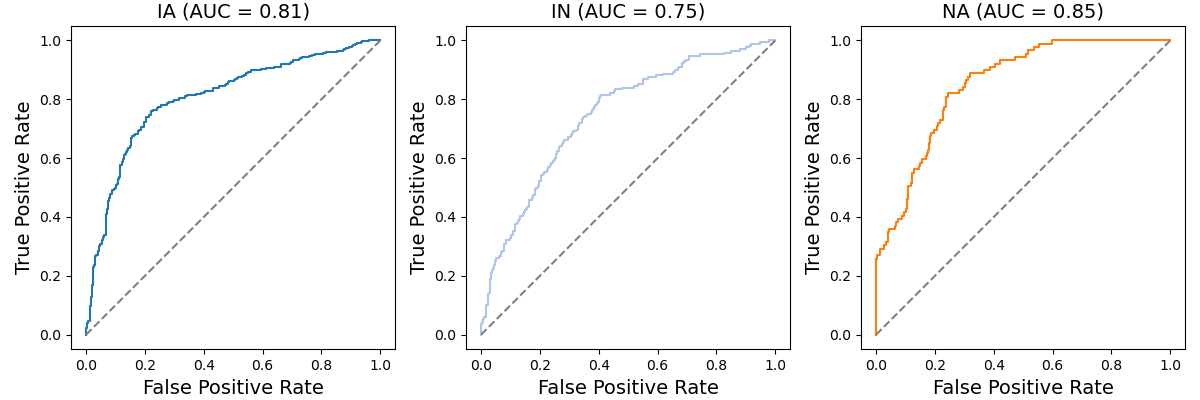
**

**Figure S7.** Internal validation visual results (I&F) – 3 ROC curves for IA, IN and NA and their corresponding AUC values.

**
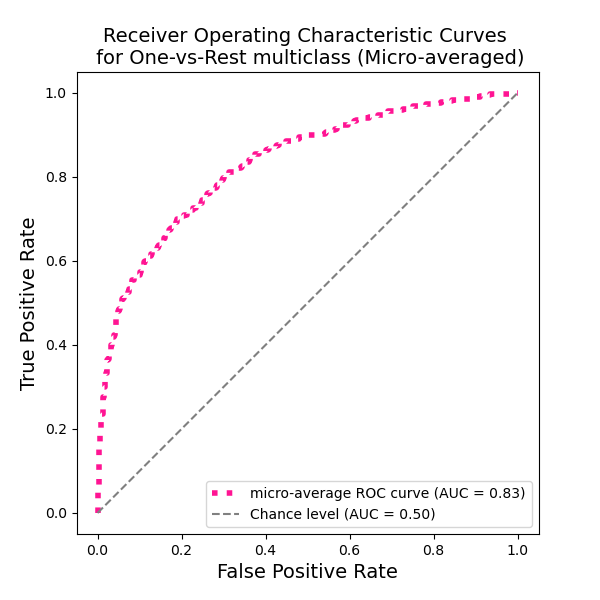
**

**Figure S8.** Internal validation visual results (I&F) – micro-average PR curve and their corresponding AP values.

**
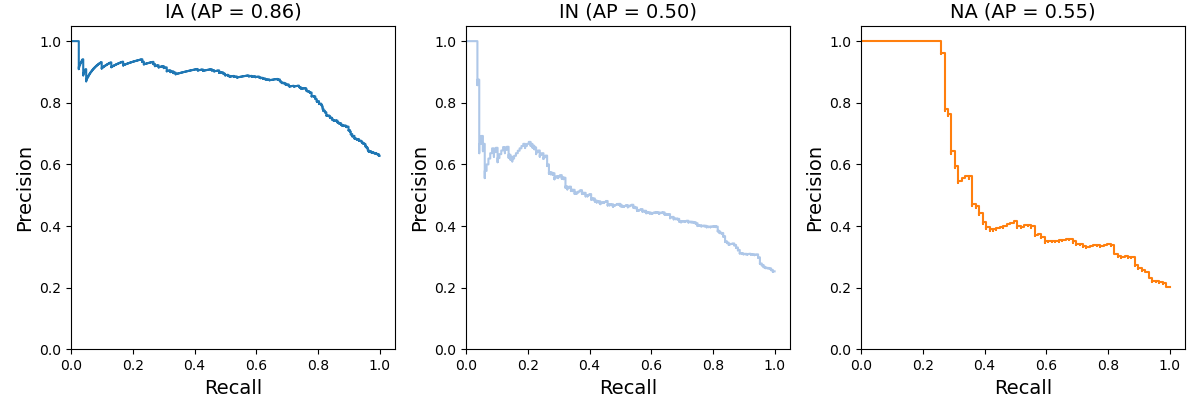
**

**Figure S9.** Internal validation visual results (I&F) – 3 PR curves for IA, IN and NA and their corresponding AP values.

**
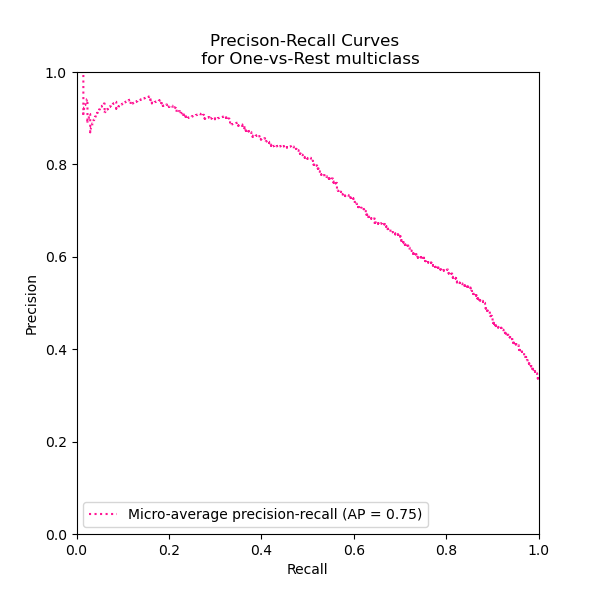
**

**Figure S10.** Internal validation visual results (I&F) – micro-average PR curve and their corresponding AP values.

2.6 External Validation (using randomly sampled labelled incident reports from 2021; n =20)

External validation aims to examine the extent to which the model performs well in an external setting. In the external validation exercise, the macro-average for the F1-score across all named entities was 83%; for the I&F task it was 50%, which is fairly similar to the internal validation exercise (see Table S4). In terms of prediction of different incident types (as a multi-label problem), the overall accuracy was 50%. We also summarise the external validation results (in terms of overall task performance, performance at the named entity level and I&F class prediction) in Figure S11.

Figure S12 (a & b) presents the AUC values across different OvR classifications for the NER tasks, ranging from 0.96 to 1. Figure S13 presents the macro-average ROC curve with AUC = 1. Figures S14 (a & b) and S15 present the PR curves for all OvR classifications, demonstrating a micro-averaged AP value of 0.98.

Figures S16 and S17 indicate the ROC curves for I&F tasks and their micro-average ROC curve, showing a micro-averaged AP value of 0.76. Figures S18 and S19 present the PR curves by class and their micro-average curve, demonstrating a micro-average AP value of 0.61.

**Table S4.** Performance evaluation: External Validation. The precision, recall and F1-score for each task are reported. Overall I&F accuracy was 0.63; class-specific scores for I&F are below.

| Marco-averaged | Precision | Recall | F1-score |
| --- | --- | --- | --- |
| NER | 0.87 | 0.80 | 0.83 |
| I&F | 0.59 | 0.47 | 0.50 |

|  | **Precision** | **Recall** | **F1-score** |
| --- | --- | --- | --- |
| **Date** | 1.00 | 0.67 | 0.8 |
| **Dosage** | 0.67 | 0.80 | 0.73 |
| **Drug** | 0.77 | 0.69 | 0.73 |
| **Duration** | 1.00 | 0.67 | 0.80 |
| **Form – form** | 1.00 | 1.00 | 1.00 |
| **Frequency** | 1.00 | 1.00 | 1.00 |
| **Route** | 0.70 | 0.74 | 0.72 |
| **Strength – amount** | 0.80 | 0.73 | 0.76 |
| **Strength –concentration** | - | - | - |
| **Strength – rate** | - | - | - |
| **Timing** | 0.91 | 0.91 | 0.91 |

|  | Precision | Recall | F1 |
| --- | --- | --- | --- |
| IA | 0.68 | 0.86 | 0.76 |
| IN | 0.47 | 0.31 | 0.37 |
| NA | 0.63 | 0.25 | 0.36 |

NER: named entity recognition; I&F: intention/factuality; IA: intended and actual; IN: intended and not actual; NA: not intended and not actual. Macro-average scores are presented. Note: the statistics were captured in a named-entity-level manner.

**Figure S11.** External validation results. **a)** Overall NER and I&F results, **b)** evaluation at the named-entity level and **c)** I&F evaluation.


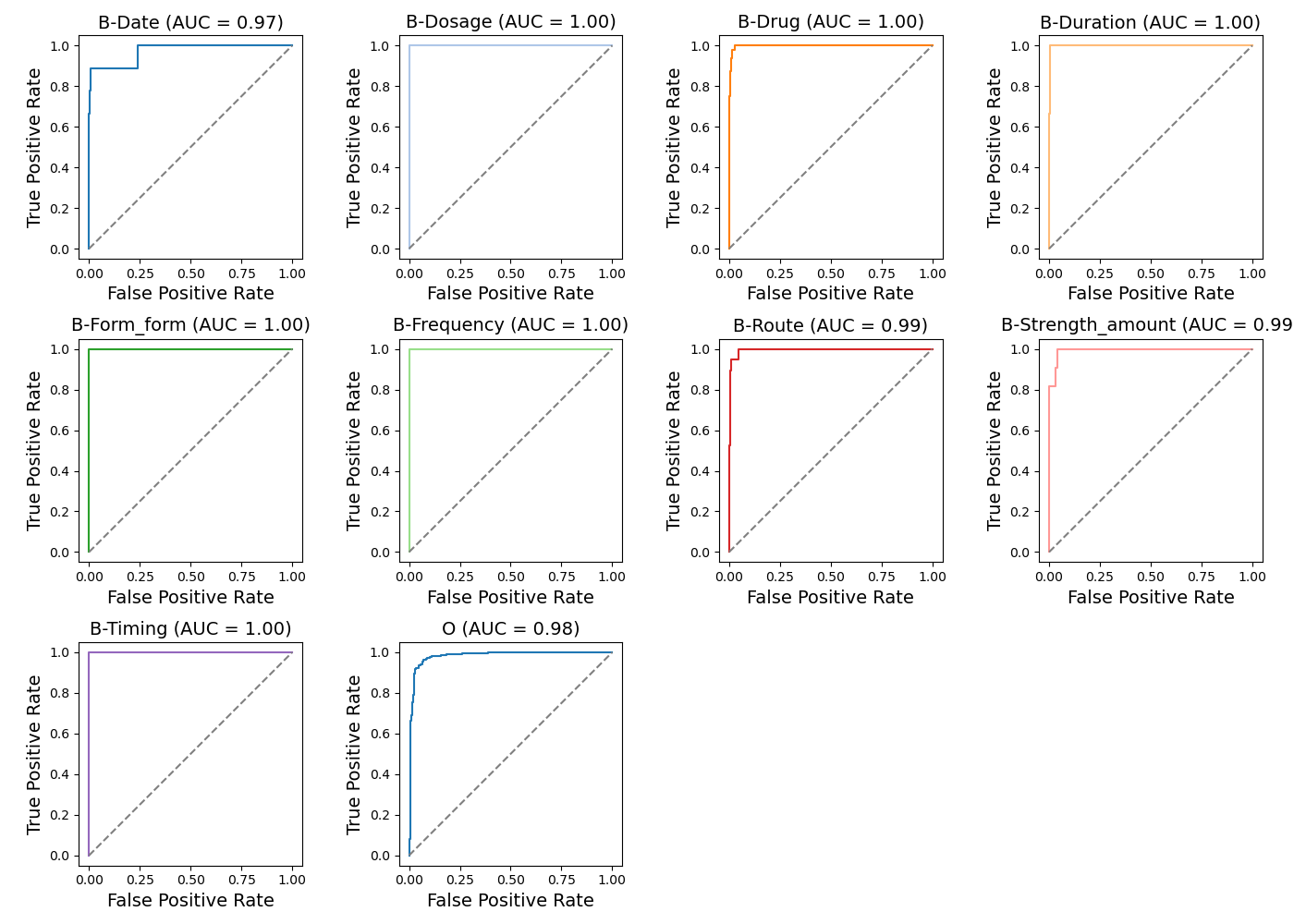


**Figure S12(a).** External validation visual results – 10 ROC curves for 9 NEs (as B) and 1 outside (as O) and their corresponding AUC values.


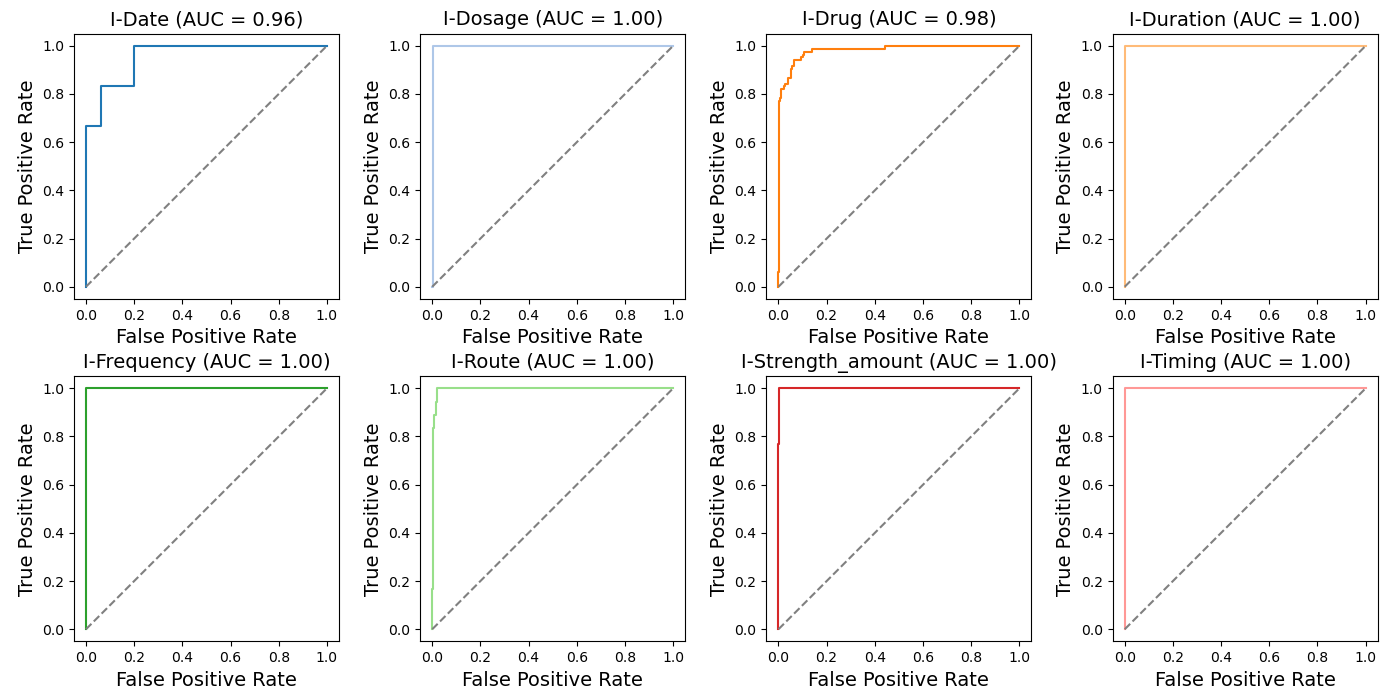


**Figure S12(b).** External validation visual results – 8 ROC curves for 8 NEs (as I) and their corresponding AUC values.


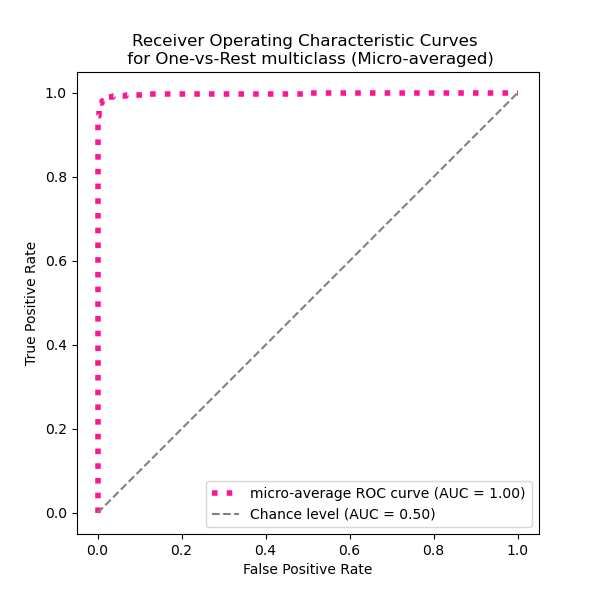


**Figure S13.** External validation visual results (NER) – micro-average ROC curve and their corresponding AUC value.


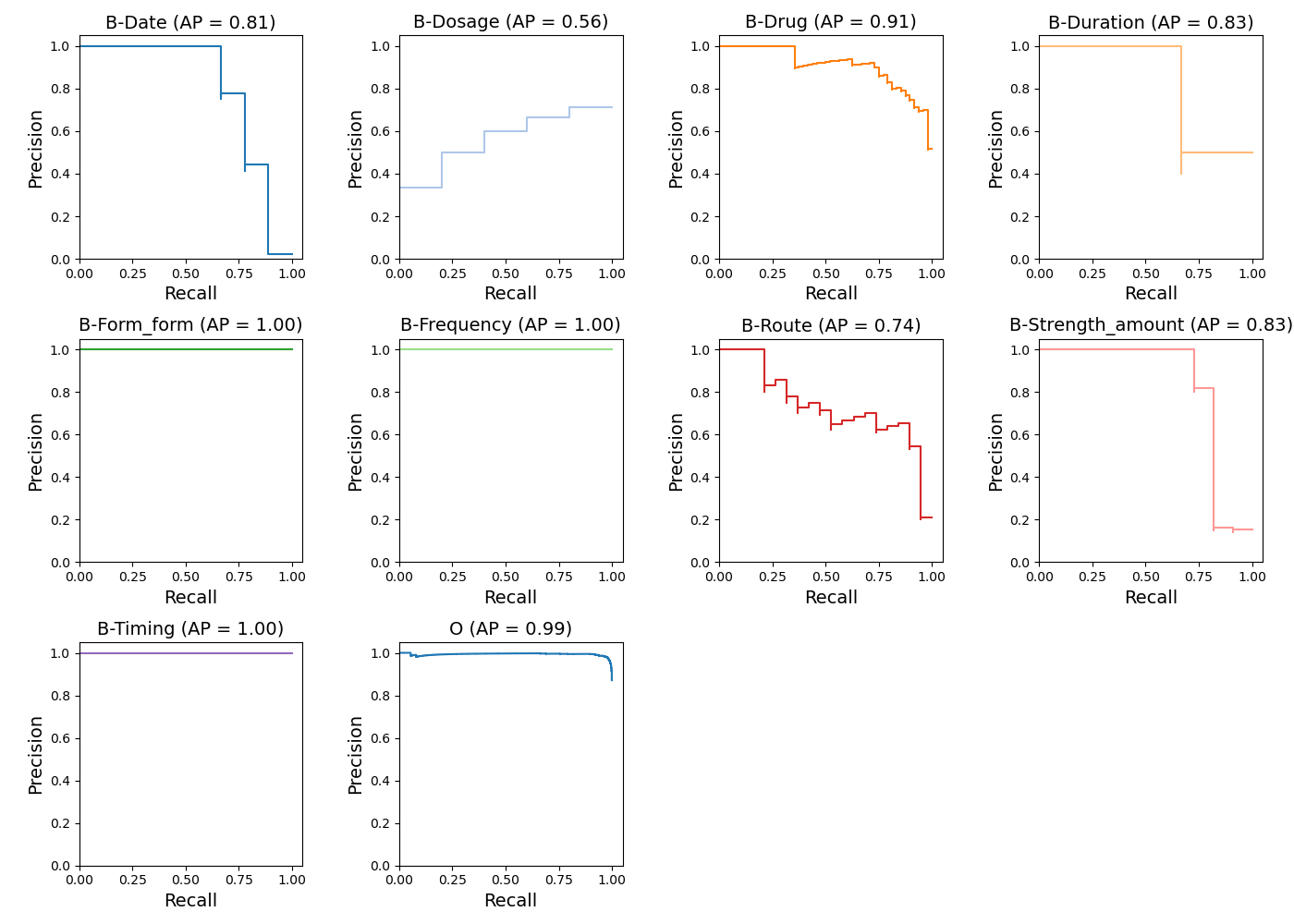


**Figure S14 (a).** External validation visual results (NER) – 10 PR curves for 9 NEs (as B) and 1 outside (as O) and their corresponding AP values.


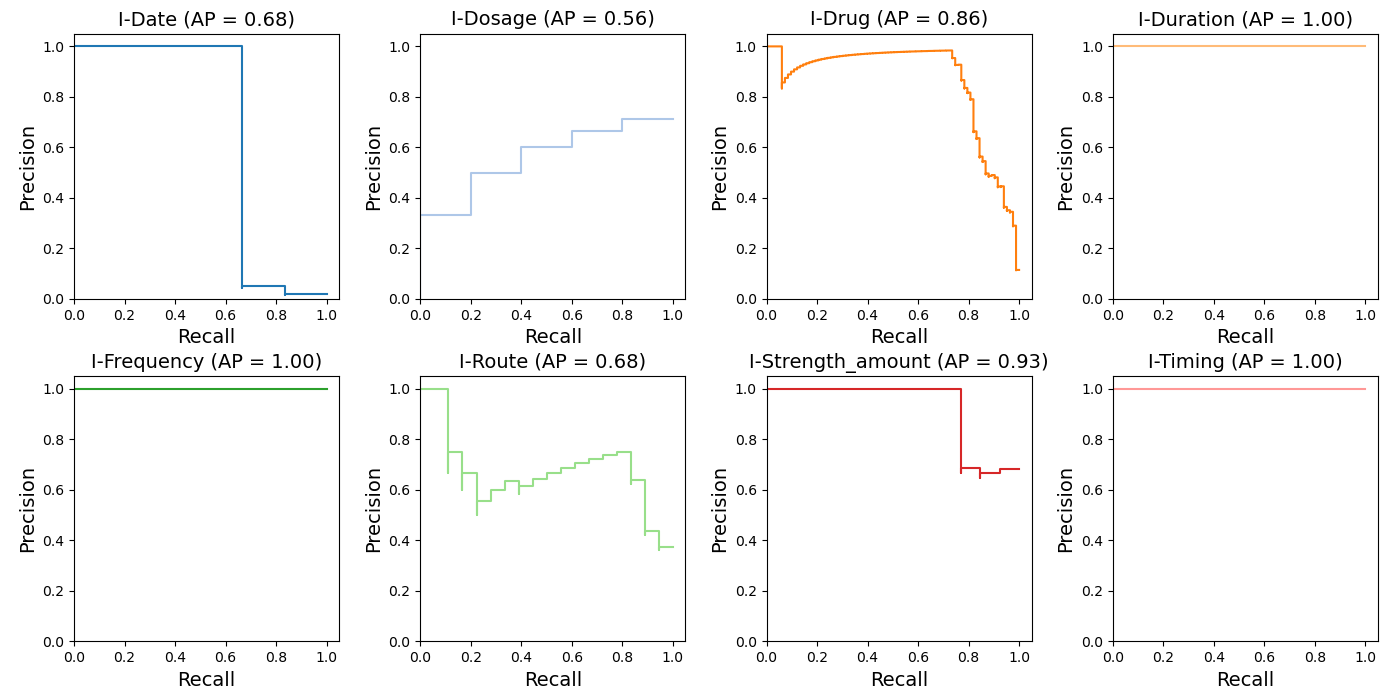


**Figure S14 (b).** External validation visual results (NER) – 8 PR curves for 8 NEs (as B) and their corresponding AP values.


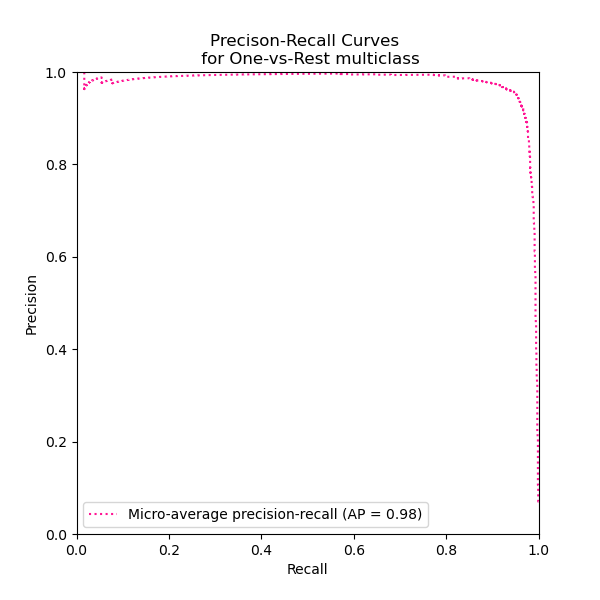


**Figure S15.** External validation visual results (NER) – micro-average PR curve and their corresponding AP values.


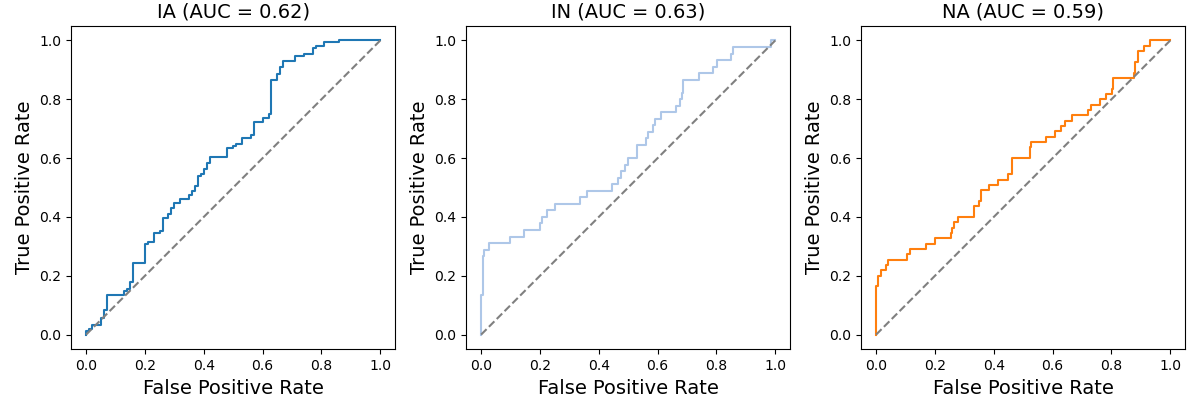


**Figure S16.** External validation visual results (I&F) – 3 ROC curves for IA, IN and NA and their corresponding AUC values.


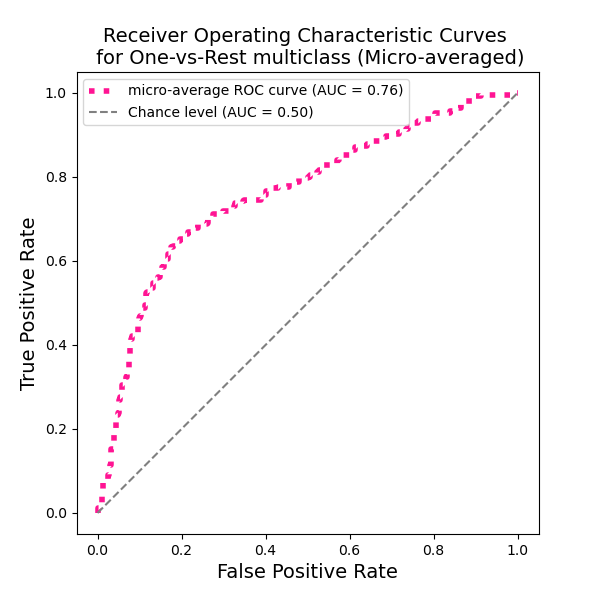


**Figure S17.** External validation visual results (I&F) – micro-average PR curve and their corresponding AP values.

**
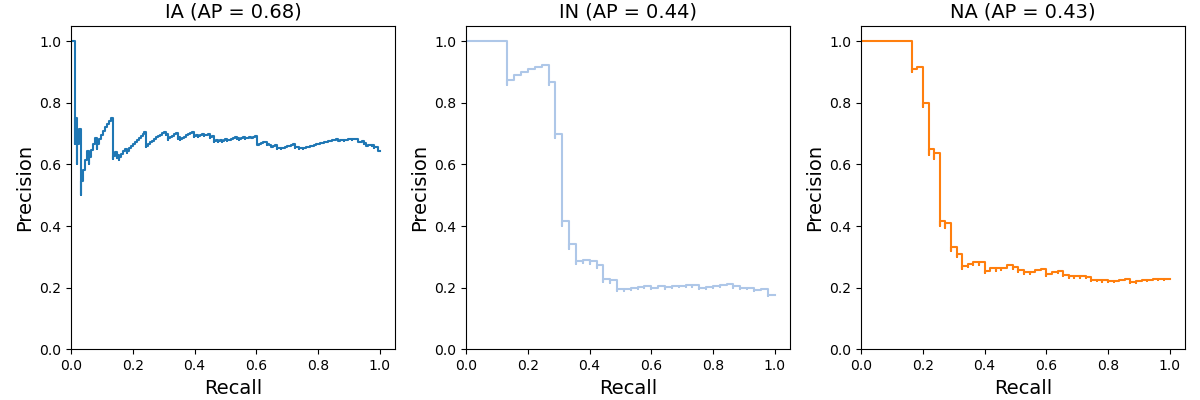
**

**Figure S18.** External validation visual results (I&F) – 3 PR curves for IA, IN and NA and their corresponding PR values.

**
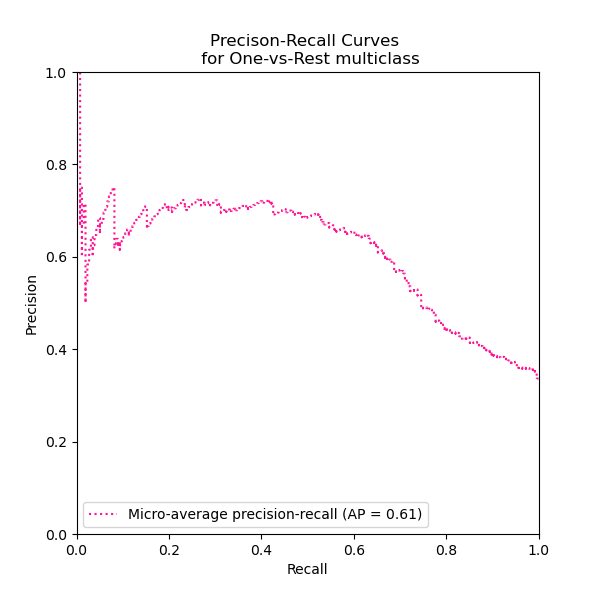
**

**Figure S19.** External validation visual results (I&F) – micro-average PR curve and their corresponding AP values.

2.7 Error Analysis (using error-free reports; n = 10)

Here we examine the robustness of the machine annotator in identifying reports without error. We input error-free reports into the machine annotator to produce prediction results. In this error analysis, the goal was to examine whether the machine annotator could categorise medication-related free texts as the incident type ‘Other’ and non-medication free texts as ‘No named entity detected’. Five reports contained medication-related named entities and five reports did not. The machine annotator correctly identified four out of five medication-related reports as ‘Other’ and for those without named entities, the machine annotator could not identify any named entities and therefore did not detect any medication-related error. In summary, the accuracy in identifying these 10 reports as “non-error” was 0.90 (9 out of 10) and the false positive rate was 0.10.

**References**

1 Japan Council for Quality Health Care. Project to collect medical near-miss/adverse event information [Iryō jiko jōhō shūshū-tō jigyō]. Japan Council for Quality Health Care. https://www.med-safe.jp/index.html (2022).

2 AI for Patient Safety Team. Annotation Guidelines for Incident Reports of Medication Errors (for English reports and for Japanese reports). AI for Patient Safety Project https://github.com/aiforpatientsafety/AnnotationGuidelines (2023).

3 Zhang, H. K., Sasano, R., Takeda, K. & Wong, Z. S. Y. Development of a medical incident report corpus with intention and factuality annotation. *LREC 2020* 4578‑4584 (2020).

4 Zhang, H. K., Sasano, R., Takeda, K. & Wong, Z. S. Y. Intention and Factuality Annotated Medical Incident Report Corpus. *GitHub* https://github.com/HongkuanZhang/IFMIR-Corpus (2020).

5 Japan Council for Quality Health Care. Project to collect medical near-miss/adverse event information [Iryō jiko jōhō shūshū-tō jigyō]. *Japan Council for Quality Health Care*<https://www.med-safe.jp/index.html> (2022).

6 Tokyo Shimbun. Information on how to apply for the Tokyo News [Tōkyō shinbun o mōshikomi no go annai]*. Tokyo Web* https://www.tokyo-np.co.jp/koudoku/k/tokyo3?gad=1&gclid=CjwKCAjw3ueiBhBmEiwA4BhspGbwa5D_4q28fpwrFDWlpm5kWGsOO53R (2023).

7 Python Software Foundation. Project description of seqeval 1.2.2. *Project seqeval* <https://pypi.org/project/seqeval/> (2023).
